# The Phospholipid Bis(monoacylglycero)Phosphate Confers Antitumour Immunogenicity to Exosomes Secreted by Dendrogenin A, Which Activates Its Biosynthesis in Tumour Cells

**DOI:** 10.1002/jev2.70225

**Published:** 2026-01-30

**Authors:** Julio Buñay, Michel Record, Philippe de Medina, Silia Ayadi, Laly Pucheu, Céline Colacios, Bruno Ségui, Marcus Höring, Gerhard Liebisch, Hélène Martin, Marc Poirot, Sandrine Silvente‐Poirot

**Affiliations:** ^1^ INOV Team “Cholesterol Metabolism and Therapeutic Innovations” Cancer Research Center of Toulouse, Equipe labellisée par la Ligue Nationale Contre le Cancer Univ Toulouse, Inserm UMR 1037, CNRS UMR 5071, CRCT Toulouse France; ^2^ Team Ceramide Metabolism In Melanoma: From Basic Mechanisms To Immunotherapy” Cancer Research Center of Toulouse Univ Toulouse, Inserm UMR 1037, CNRS UMR 5071, CRCT Toulouse France; ^3^ Institute of Clinical Chemistry and Laboratory Medicine University Hospital Regensburg Regensburg Germany; ^4^ Institute for Infectious and Inflammatory Diseases Univ Toulouse, INSERM, CNRS, Infinity Toulouse France

**Keywords:** anti‐PD‐1, LXR, dendrogenin A, BMP, LBPA, CLN5, Phospholipase D

## Abstract

Dendrogenin A (DDA) is a cholesterol‐derived antitumour metabolite that promotes the secretion of immunogenic antitumour exosomes (DDA‐sEV) enriched in bis(monoacylglycero)phosphate (BMP). BMP is a phospholipid specific to late endosomes and lysosomes, where it plays a crucial role in lipid degradation, regulates the fate of endosomal cholesterol, and contributes to intraluminal vesicle formation. Dysregulation of BMP biosynthesis is associated with multiple diseases. Here, we show that the DDA/LXRβ complex activates the transcription and activity of phospholipase D (PLD) and CLN5, two enzymes involved in BMP biosynthesis. Inhibition of PLD in DDA‐treated tumour cells reduces BMP levels in DDA‐sEV, impairs their release, and their antitumour immune activity. Blocking BMP on DDA‐sEV with a specific antibody abolishes their antitumour reponse, prevents the recruitment of activated dendritic cells (DC) and T cells into tumours, and decreases mouse survival. This blockade also impairs DDA‐sEV uptake by immature DC (iDC) and hinders DC maturation and Th1 T cell activation. Notably, neutralising the BMP‐presenting receptor on iDC inhibits DDA‐sEV uptake and DC maturation. Treatment of iDC with free BMP induces their functional maturation, confirming BMP as a key immune activator. Furthermore, BMP‐containing DDA‐sEV enhance the efficacy of anti‐PD‐1 therapy in melanoma. Targeting LXRβ with DDA represents an innovative strategy to stimulate anticancer immunity by increasing BMP levels in tumours and sEV.

## Graphical abstract

Dendrogenin A stimulates, via LXRβ, the expression and activity of enzymes responsible for the biosynthesis of the phospholipid BMP. BMP is a key determinant of the antitumour immunogenicity of exosomes secreted by DDA‐treated melanoma and triple‐negative breast cancer cells and enhances mouse survival and anti‐PD‐1 therapy in melanoma.

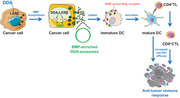

## 1 Introduction

Exosomes are phospholipid bilayer vesicles of 30–150 nm in diameter that are secreted by all types of immune and non‐immune cells and enable the exchange of materials between cells (Record et al. 2011, Mathieu et al. 2019). Exosomes are part of small extracellular vesicles (sEV); they are formed inside late endosomes or multivesicular bodies (MVB) by the inward budding of the limiting membrane, which generates intraluminal vesicles (ILV). Fusion of MVB with the plasma membrane results in the secretion of ILV termed “exosomes”. sEV (exosomes) originating from MVB contain diverse proteins, lipids and nucleic acids representative of the parental cells and their content reflects selective sorting processes operating within cells. Their uptake by target cells can induce specific biological functions that depend on the components loaded into sEV and subsequently transferred to recipient cells. sEV secreted from normal cells play beneficial roles in tissue integrity and immunosurveillance, whereas sEV constitutively secreted by tumour cells (tumour‐sEV) contribute to promote tumour progression, aggressiveness and immunosuppression (Pitt et al. 2016, Peinado et al. 2012, Robbins and Morelli 2014, Hoshino et al. 2015, Whiteside 2016, Li and Nabet 2019, Ortiz et al. 2019, Han et al. 2022, Kalluri and McAndrews 2023). These findings highlight the therapeutic relevance of pharmacologically controlling the content of tumour sEV to stimulate anti‐tumour immunity. We previously reported that this can be achieved using dendrogenin A (DDA), an endogenous cholesterol metabolite and a modulator of the liver X receptor α and β (LXRs) (De Medina et al. 2013, Segala et al. 2017, Record et al. 2022, de Medina et al. 2023, Poirot et al. 2025). LXRs are nuclear receptors and ligand‐dependent transcription factors, that regulate lipid metabolism and immunity (Segala et al. 2017, Record et al. 2022, Poirot et al. 2025, Lin and Gustafsson 2015, Pontini and Marinozzi 2020, Silvente‐Poirot et al. 2018, Silvente‐Poirot et al. 2018, Bosteels et al. 2023, Yan et al. 2023). In melanoma and breast cancer cells, we showed that the DDA/LXRβ complex increases the secretion of exosome‐enriched sEV, termed DDA‐sEV. This was associated with increased levels of the phospholipid bis(monoacylglycero)phosphate (BMP) in tumour cells and in DDA‐sEV (Record et al. 2022). DDA‐sEV significantly inhibit tumour growth in immunocompetent mice and promote the maturation of human dendritic cells (DC) which activate Th1 T cell polarization *ex‐vivo* (Record et al. 2022). BMP, previously termed lysobisphosphatidic acid (LBPA), accumulates in the internal membranes of MVB and ILV (Kobayashi et al. 1998, Möbius et al. 2003). BMP is a fusogenic lipid at the acidic pH of endosomes (Kobayashi et al. 2002). Importantly, it promotes ILV formation when added to liposomes (Matsuo et al. 2004). Consistent with its involvement in sEV formation, decreasing BMP level in tumour cells by genetic invalidation of the LXRβ decreased DDA‐sEV secretion (Record et al. 2022). BMP is also involved in the sorting and trafficking of specific proteins and lipids in late endosomes (Kobayashi et al. 2002). Furthermore, BMP plays a crucial role in lipid degradation and in regulating the fate and levels of endosomal cholesterol (Chevallier et al. 2008). Dysregulation of BMP levels has been implicated in several lysosomal lipid storage diseases, such as Niemann–Pick disease type C, and in anti‐phospholipid syndrome (Gruenberg 2020). Alterations in BMP levels lead to sphingolipid and cholesterol storage disorders and ganglioside accumulation resulting in inflammation and neurodegeneration (Boland et al. 2022, Medoh et al. 2023, Singh et al. 2024). However, its role in cancer remains unknown. Recent major advances have identified that BMP can be presented by the endothelial protein C receptor (EPCR) which is present at the surface of myeloid cells (Müller‐Calleja et al. 2021), and that the enzymes involved in BMP biosynthesis include CLN5 (Medoh et al. 2023) and phospholipases D 3/4 (Singh et al. 2024). In addition, the lysosomal PLA2G15 was shown to produce LPG, a precursor of BMP (Chen et al. 2023) or to hydrolyse some BMP isomers (Nyame et al. 2025). In the present study, we investigated the role of BMP in the anti‐tumour immunogenicity of DDA‐sEV and the mechanism by which DDA increases BMP biogenesis in tumour cells and BMP levels in DDA‐sEV. We showed that BMP plays a critical role in DDA‐sEV secretion and anti‐tumour immune responses and sensitizes melanoma to anti‐PD‐1 therapy. Moreover, we established that the DDA/LXRβ complex stimulates the expression and activity of the enzymes that regulate BMP biosynthesis.

## 2 Materials and Methods

### 2.1 Reagents

PLD1 inhibitor‐VU0359595 Cat # 857371P, DMSO (dimethyl sulphoxide) Cat # 20–139, propidium iodide Cat # P4864, EDTA Cat # E7889 and 5(6)‐carboxyfluorescein diacetate *N*‐succinimidyl ester (CFSE) Cat # 21888, were from Sigma‐Aldrich, France. BMP (18:1 BMP S,S) was from Avanti Polar lipids, Cat # 857135P. Phosphatidylcholine (PC) was from MedChemExpress, Cat # HY‐125853. DDA was synthesised as previously described (Segala et al. 2017) and was confirmed to be 99% pure by LC/MS.

### 2.2 Tumour Cell Culture

The SKMEL28 cell line is a malignant melanoma of human origin, BRAF V600E mutated, obtained from (LGC standards, France). ShLXRβ SKMEL28 and shC SKMEL28 cells were obtained as described in (Segala et al. 2017). B16K1 was provided by Dr. Bruno Segui (Montfort et al. 2021). TNBC 4T1 cells were from ATCC (CRL‐2539). Cells were kept at 37°C in 95% air and 5% CO_2_. All culture reagents were from Gibco, France, except foetal bovine serum (FBS) from Dutscher. SKMEL28 cells were grown in RPMI medium 1640 supplemented with 2 mM l‐glutamine (Cat # 25030024), 10% FBS previously heat‐inactivated at 56°C for 1 h and penicillin and streptomycin (50 units/mL). France. B16K1 cells were grown in DMEM 4 g/L sucrose medium (Cat # 41966) supplemented with 1 mg/mL G418 (Sigma‐Aldrich, Cat # G8168), 10 % FBS, penicillin, streptomycin (50 units/mL) (Cat # P0781) and 2 mM l‐glutamine (Cat # 25030024). 4T1 cells were grown in RPMI medium 1640 (Cat # 21875) supplemented with 10% FBS, penicillin and streptomycin (50 units/mL) and 2 mM l‐glutamine (Cat # 25030024). All cell lines were used within 20 passages following thawing and tested monthly and before injection into mice, for mycoplasma contamination using Mycoalert Detection kit (Lonza, Cat # 10255393). For all treatments, cells were seeded at 60%–70% confluence.

### 2.3 sEV Preparation

sEV preparation was performed as reported by (Record et al. 2022). Briefly, tumour cells were seeded in 150 mm culture disks (Corning, Cat # 353025) at 2.4×10^6^ cells in 20 mL complete culture medium. The medium was removed 24 h later and replaced by the same medium supplemented with EV‐free FBS, obtained by overnight ultracentrifugation at 110 000 g at 4°C and passed through a 0.2 µm filter. B16K1 and 4T1 cells were treated for 24 h with 2 or 1 µM of DDA, respectively, or 1/1000 ethanol as solvent vehicle. sEV preparation was carried out under sterile conditions using sterilisable ultracentrifuge tubes (Beckman Coulter, France). sEV were recovered from the culture medium and processed by differential centrifugations as described (Record et al. 2022, Subra et al. 2010). Then, the supernatant was recovered and sEV were then pelleted at 110 000x g for 70 min, resuspended in 20 mL PBS and centrifuged again at 110 000 x g for 70 min. Finally, sEV pellet was diluted in around 150–200 µL calcium free‐PBS. The sEV used were endotoxin‐free as verified using a LAL chromo endotoxin quantitation kit (Thermo‐Fisher Scientific, France).

### 2.4 Protein Quantification

sEV protein content was quantified by the colorimetric method of Lowry in the presence of 0.1% w/v SDS (Record et al. 2022, Subra et al. 2010). Proteins from tumour cells were extracted using a RIPA buffer (Sigma‐Aldrich, Cat # R0278) supplemented with complete protease and phosphatase inhibitor cocktail, respectively (Sigma‐Aldrich, France, Cat # P8340 / P5726 / P0044). Then, proteins were purified by centrifugation at 12 000 × g at 4°C for 30 min and subsequently quantified by BCA protein assay (Thermo Scientific, Cat # 23227).

### 2.5 Characterization of sEV by Immune‐Blot Analysis

sEV (7 µg) or cells extract (20 µg) were diluted in sample buffer and denaturated by heating at 70°c for 10 min. Identical amounts of proteins were loaded and separated by electrophoresis in polyacrylamide gels (SDS–PAGE) under denaturing and reducing conditions and transferred onto PVDF membranes. Membranes were blocked with a solution of 5% (w/v) non‐fat milk in TBS‐Tween 0.1% or BSA, 0.1% Tween in PBS, pH 7, and incubated overnight with the indicated specific primary antibodies: anti‐LC3 (Merck/Sigma‐Aldrich, France, L8918, 1/2000), anti‐MFGE8 (Merck/Sigma‐Aldrich, France, SAB1408603, 1:1000), anti‐CD63 (Abcam, United Kingdom, ab217345, 1/1000), anti‐HSP70 (Abcam, United Kingdom, ab45133, 1/2000), anti‐Melan A (Abcam, United Kingdom, ab210546, 1/2000), anti‐TRP2 (Santa‐Cruz, Germany, sc‐74439, 1:/500), anti‐ALIX (Santa‐Cruz, Germany, sc‐271975, 1/500) (Table S2). Detection was performed using a peroxidase‐conjugated anti‐rabbit IgG (Promega, France, Cat # W4011) or anti‐mouse IgG (Ozyme, France, Cat # 7076S) diluted 1/5000 in blocking solution for one hour at room temperature. Proteins were revealed using the chemiluminescence kit Clarity Western ECL Blotting Substrates (Bio‐Rad, Cat # 170–5061) and analysed by ChemiDoc imager (Bio‐Rad).

### 2.6 Characterization of sEV Content by Flow Cytometry Analysis

sEV (7 µg) were incubated with 7 µL of 4 µm aldehyde‐sulphate latex beads (Thermo‐Fisher Scientific, cat #A37304, France) in 200 µL PBS overnight at 4°C under shaking at 300 rpm. Free sites on latex beads were next saturated with 200 µL solution PBS–BSA 4% for 30 min at 25°C under shaking. Beads with bound sEV were centrifuged for 5 min at 5000 rpm, washed in 1 mL PBS, and diluted in 200 µL FACS buffer (PBS containing 2% heat‐inactivated FBS and 1 mM EDTA). Beads with bound sEV were incubated overnight at 4°C with gentle shaking with the indicated specific primary antibodies (Table S2) in the following proportion: anti‐BMP (Sigma‐Aldrich, France, Cat # MABT837, clone 6C4) at a ratio of 0.5 µg per µg of sEV, anti‐CD63 (Santa‐Cruz, Germany, Cat # sc5275) at a ratio of 1 µg per µg of sEV, and anti‐PD‐L1 Euromedex, France, Cat # BX‐BE0101, clone 10F.9G2) at a ratio of 1 µg per µg of sEV; or with the isotypes controls anti‐IgG1 (Sigma‐Aldrich, France, Cat # CBL610, clone 1E2.2), anti‐IgG1 (Santa‐Cruz, Germany, Cat # sc3877) or anti‐IgG2b (Euromedex, France, Cat # BE0090, clone LTF‐2) at the same ratio than the corresponding primary antibody. After centrifugation and washing, dye‐coupled AF488‐conjugated secondary antibody: anti‐mouse (Thermo Fisher Scientific, France, Cat # A‐11001, 1/50) or anti‐rat (Thermo Fisher Scientific, France, Cat # A48262, 1/50) was added and incubated for 1 h at 4°C. Beads with bound antibody‐labelled sEV were diluted in FACS buffer and analysed by flow cytometry using MACSQuant 10 analyser (Miltenyi Biotec). 50 000 events were analysed from each sample, followed by Flow‐Jo software analysis.

### 2.7 Blockade of CD63, PD‐L1 and BMP on sEV With Specific Antibodies

Purified sEV (200 µg) were first saturated with 200 µL of PBS‐BSA 4% BSA for 30 min at 4°C. Then, sEV were incubated 2 h at 4°C with the following specific primary antibody: anti‐BMP (100 µg/mL) (Sigma‐Aldrich, France, Cat # MABT837, clone 6C4) at a ratio of 0.5 µg of anti‐BMP per µg of sEV, anti‐CD63 (200 µg/mL) (Santa‐Cruz, Germany, Cat # sc5275) at a ratio of 1 µg of anti‐CD63 per µg of sEV, and anti‐PD‐L1 (10 µg/mL) (Euromedex, France, Cat # BX‐BE0101, clone 10F.9G2) at a ratio of 1 µg of anti‐PD‐L1 per µg of sEV; or the isotypes controls used at the same ratio than the corresponding primary antibody: anti‐IgG1 (Sigma‐Aldrich, France, Cat # CBL610, clone 1E2.2), anti‐IgG1 (Santa‐Cruz, Germany, Cat # sc3877) or anti‐IgG2b (Euromedex, France, Cat # BE0090, clone LTF‐2). Next, each sEV preparation was resuspended in 20 mL PBS and ultracentrifuged at 110 000 x g for 70 min to remove non‐bound free antibodies. Finally, sEV pellet was diluted in calcium free‐PBS and the sEV proteins were re‐quantified by the Lowry method as described below. Blocking sEV were either incubated with iDC or injected in mice as indicated in the corresponding paragraph. For in vivo experiments, the indicated free antibodies, used as controls, were tested at the same concentration/volume than those used for sEV blocking. For ex vivo iDC maturation experiments, free anti‐BMP was tested at 1 µg/mL.

### 2.8 CFSE‐Labelling of sEV

sEV were purified as described above in the “sEV preparation” paragraph. After the first ultracentrifugation, sEV pellet was resuspended in 20 mL of medium of culture containing 1 µL of carboxyfluorescein diacetate N‐succinimidyl ester (CFDA‐SE) at 20 µM and incubated 15 min at 37°C. CFDA‐SE is a membrane‐permeable compound that fluoresces after cleavage by esterases present in the lumen of EV, thus forming CFSE which covalently binds to primary amines inside sEV (Loconte et al. 2023). To remove free CFDA‐SE, the sEV was then centrifuged at 110 000 x g for 70 min at 4°C. The sEV pellet was washed again with 20 mL PBS and centrifuged at 110 000 x g for 70 min. CFSE‐labelled sEV pellets were diluted in calcium free‐PBS and stored at ‐80°C until use.

### 2.9 Nano Particle Tracking Analysis (NTA)

NTA was performed using NanoSight (NanoSight NS300, Malvern Panalytical, Malvern, UK). sEV preparations were diluted 1/1000 in filtered PBS (0.2 µm) and monitored using a NanoSight NS300 (Malvern Panalytical) equipped with a 405 nm laser. Videos were recorded three times during 60 s for each sample at constant temperature (22°C) and analysed with NTA Software 3.4 (Malvern instruments Ltd).

### 2.10 Generation of Mouse iDC *In Vitro*

Bone marrow‐derived cells (BMDC) were isolated from the tibias and femurs of C57B/L6 mice (Lutz et al. 2023). Briefly, BMDC were suspended in RPMI 1640 medium (Gibco, France, Cat # 21875), supplemented with penicillin and streptomycin (50 units/mL) and kept on ice. Red blood cells were depleted using RBC Lysis buffer (BioLegend, France, Cat # 420301) and filtered through a 70 µm filter. Then, cells were cultured in 150 mm culture disks (Corning, Cat # 353025) at 3.0×10^6^ cells in 20 mL in RPM1 1640 medium supplemented with 2 mM l‐glutamine, 10% FBS heat‐inactivated at 56°C for 1 h, penicillin and streptomycin (50 units/mL), 1 mM Hepes (Gibco, France, Cat # 15630‐056), 1% non‐essential amino acids (100×) (Gibco, France, Cat # 11140‐050), 50 µmol/L 2‐Mercaptoethanol (Gibco, France, Cat # 31350‐010) (designated “iDC culture medium”), and 20 ng/mL GMCSF (Peprotech, France, Cat # 315‐03‐100UG), at 37°C in a humidified atmosphere with 5% CO2. iDC culture medium containing 20 ng/mL GMCSF was refilled every 3 days. iDC are obtained on day 6 of culture.

### 2.11 Blockade of EPCR on iDC

iDC were generated as described above. iDC (1×10^6^) were recovered by centrifugation at 400 x g for 5 min at 4°C and first saturated with 200 µL of PBS‐BSA 4% for 30 minutes at 4°C. Next, cells in 4 mL “iDC culture medium” were incubated for 1 h with gentle agitation at 37°C with the indicated antibodies at 2.5 µg/mL: Anti‐CD201 (EPCR) (Thermo Fisher Scientific, France, Cat #16‐2012‐83, clone eBio1560) or the rat IgG2b kappa isotype control (Thermo Fisher Scientific, France, Cat #16‐4031‐85, clone eB149/10H5) (Table S2). At the end of the incubation period, anti‐EPCR‐iDC or anti‐IgG2b‐iDC were harvested by centrifugation at 400 x g for 5 min at 4°C, washed twice with 3 mL of PBS to eliminate unbound antibodies and counted and maintained in iDC culture medium.

### 2.12 Analysis of CFSE‐sEV Uptake by iDC

The indicated iDC (iDC, anti‐EPCR‐iDC and anti‐IgG2b‐iDC), generated as described above, were cultured in 4 mL of iDC culture medium (0.75×10^6^ cells in T25 flask) and were incubated with CFSE‐labelled C‐sEV or DDA‐sEV (5 µg/mL) at the indicated times and at 37°C or 4°C as indicated in the figure legend. In addition, to distinguish true uptake from passive adsorption, we investigated the incorporation of the indicated CFSE‐labelled‐sEV into the indicated iDC for 2 h at 4°C. At the endpoints, the indicated iDC were harvested by centrifugation at 400 x g for 5 min at 4°C and washed twice with 2 mL of PBS before analysis by flow cytometry (FACS) or confocal microscopy. For FACS analysis: the indicated iDC were recovered and stained with LIVE/DEAD reactive dye (Thermo Fisher Scientific, France, Cat # L34966A, 1:300) and anti‐mouse CD11c (BD Bioscience, France, Cat # 553802, clone HL3, 1:200), Table S2, for 30 min at 4°C in FACs buffer. Next, samples were washed twice with 2 mL PBS. CFSE‐labelled‐sEV uptake by the indicated iDC was measured by flow cytometry using MACSQuant 10 analyser (Miltenyi Biotec, France), 20 000 events were analysed from each sample, followed by Flow‐Jo software analysis, gating strategies are depicted in supplementary data files. For confocal microscopy of sEV uptake by iDC: iDC were recovered and stained with an anti‐mouse CD11c (BD Bioscience, France, Cat # 553802, clone HL3, 1/200) for 30 min at 4°C. Cells were then washed twice, and were fixed with paraformaldehyde at 4% in PBS. Slides were mounted with Prolong Gold antifade reagent containing Dapi (Invitrogen, Cat # P36941) covered with a glass coverslip, sealed, and stored in the dark at 4°C. Images were acquired with Zeiss LSM 800 AiryScan (z‐stack image) at 63X magnification. All images were analysed using Fiji software.

### 2.13 sEV and Lipid Treatment of iDC

The indicated iDC (iDC, anti‐EPCR‐iDC and anti‐IgG2b‐iDC) generated as described above were recovered by centrifugation at 400 x g for 5 min at 4°C. Then, the indicated iDC (0.75×10^6^ cells/T25 flask) were cultured in 4 mL of iDC culture medium without GM‐CSF for 24 h at 37°C, with the indicated sEV (5 µg/mL), or the solvent vehicle (1/1000 ethanol) or increasing concentrations of free BMP (0.1, 1, 5, 10 and 20 µM) or 20 µM free PC, or LPS (1 mg/mL). BMP was not incorpored in liposomes or micelles as reported by (Müller‐Calleja et al. 2021), instead, it was solubilised in ethanol/PBS (1/1000) as was PC. At the endpoint, the cells were harvested and analysed as indicated.

### 2.14 Analysis of DC Maturation and Intracellular Cytokines Expression by Flow Cytometry

Analysis of iDC maturation: At the endpoint, iDC were harvested by centrifugation at 400 x g for 5 min at 4°C and then incubated for 20 min at 4 °C with Fc block antibody anti‐CD16/CD32 (Thermo Fisher Scientific, France, Cat # 14‐0161‐85, clone 93, 1/200) in FACS buffer. Next, cells were stained with LIVE/DEAD reactive dye (Thermo Fisher Scientific, France, Cat # L34966A) and labelled for membrane markers with the following antibodies (Table S2): anti‐CD11c (Cat # 130‐110‐840, clone REA754, 1/200), anti‐MHC‐II (Cat # 130‐112‐388, clone REA813, 1/200), anti‐CD80 (Cat # 130‐116‐465, clone REA983, 1/200), anti‐CD86 (Cat # 130‐122‐129, clone REA1190, 1/200), from Miltenyi Biotec, France, and anti‐CD197 (CCR7) (Thermo Fisher Scientific, France, Cat # 47‐1971‐82, clone 4B12, 1/100) or controls isotypes used at the same ratio than the corresponding primary antibody (Table S2) in FACS buffer at 4°C for 30 min. Next, samples were washed twice with 2 mL of PBS.

Analysis of intracellular cytokines in iDC: After treatments with the indicated sEV (5 µg/mL) for 24 h, cells were treated with 3 µg/mL of BrefeldinA (Thermo Fisher Scientific, France, Cat # 00‐4506‐51) at 37°C for 4 h. Then, cells were recovered and pre‐incubated with Fc block antibody anti‐CD16/CD32 as described above. Samples were stained with LIVE/DEAD reactive dye and labelled with the following antibodies: anti‐CD11c (Cat # 130‐110‐840, clone REA754, 1/200) and anti‐MHC‐II (Cat # 130‐112‐388, clone REA813, 1/200) from Miltenyi Biotec, France, in FACS buffer for 30 min at 4°C. Then, cells were fixed and permeabilised using fixation/permeabilisation staining buffer set (Thermo Fisher Scientific, France, Cat #00‐5523‐00) following the manufacturer´s instructions, washed with PBS and incubated overnight, in FACS buffer at 4°C, with anti‐TNF‐α (Cat # 130‐124‐212, clone REA636, 1/50) and anti‐IL‐12 (p40/p70) (Cat # 130‐102‐163, clone REA136, 1/50) or controls isotypes from Miltenyi Biotec, France (Table S2). Next, samples were washed 3 times with 2 mL PBS. All samples were analysed using an LSRFortessa X‐20 (BD Biosciences), and data were analysed using FlowJo^TM^ v10 software (BD Life Sciences). Gating strategies are depicted in supplementary data files.

### 2.15 Gene Expression Analysis by RT‐qPCR

Total RNA from the indicated cells was extracted using Trizol Reagent (Thermofisher Scientific, France, Cat # 10296028) according to the manufacturer's instructions. RNA was diluted with RNase‐free water and the concentration was measured on a ND‐1000 spectrophotometer (NanoDrop). Reverse transcription was performed using 1 µg RNA iScript Reverse Transcriptase (Bio‐Rad, France, Cat # 170–8891) in a final reaction volume of 20 µL diluted in RNase‐free water on an Eppendorf Mastercycler (Eppendorf, France). Real‐time quantitative qRT‐PCR analysis was performed using 1 µL of 1:5 diluted cDNA template and SyBR Supermix (Bio‐Rad, France, Cat # 170–8884). PCR profiles were obtained using LightCycler 480 system (Roche Diagnostics, France). Relative gene expression was obtained using the 2^−ΔΔCt^ method (Schmittgen and Livak 2008). Data performed in mice were normalised with *RPLP0* (*36b4*) and GAPDH, and in human with *RPLP13 and GAPDH*. All primers are listed in Table S3.

### 2.16 Immunochemistry and Immunofluorescence Analysis

Immunochemistry analysis of tumours: Tumours were fixed in 10% neutral buffered formalin, paraffin‐embedded, and histological sections of 5 µm thickness were mounted on slides. Briefly, slides were deparaffinised and rehydrated by standard protocol. Antigen retrieval was performed by heated until boiling for 20 min in sodium citrate 10 mM pH 6.0. Samples were treated with 0.3% (v / v) H_2_O_2_ for 10 min, followed by 30 min of protein blocking using a solution of 1% BSA to prevent non‐specific binding. Then, slides were incubated overnight at room temperature, with the indicated specific primary antibodies (Table S2): anti‐CD3 (Dako, France, Cat # RBK024, 1/100), anti‐CD4 (Cell Signalling, France, Cat # D7D2Z, 1/100) and anti‐CD8 (Cell Signalling, France, Cat # D4W2Z, 1/500) and anti‐CD11c (Tebu‐bio, France, Cat # CL8923AP, clone N418, 1/100). Detections were performed using the NovaRED substrate kit for peroxidase (Vector Laboratories, Cat # SK‐4805,). Nuclei were counterstained with haematoxylin and slides were mounted in PBS‐glycerol covered with a glass coverslip, sealed, and stored in the dark at 4°C until imaged. Immunofluorescence analysis of tumour cells and iDC: At the endpoint, tumours cells cultured on glass coverslips or iDC harvested by centrifugation at 400 x g for 5 min at 4°C, were fixed in 4% paraformaldehyde in PBS, saturated with 1% BSA‐1% BSA and permeabilised 0.1% tritonx100 in PBS. Cells were incubated with the specific primary antibody anti‐BMP (Sigma/Merck, Cat # MABT837 clone 6C4, 1/200) overnight. Then cell were washed twice and incubated with dye‐coupled AF488‐conjugated anti‐mouse secondary antibody (Thermo Fisher Scientific, France, Cat # A‐11001, 1/500), for 1 h at 25°C. Then washed five times with 1% BSA‐0.1% TritonX100 PBS.

Non‐fixed and permeated iDC were recovered and directly incubated with the primary antibody anti‐CD201 (EPCR) (Thermo Fisher Scientific, France, Cat #16‐2012‐83, clone eBio1560, 1:100) for 30 h at room temperature, then iDC washed five times with 1% BSA‐0.1% Tritonx100 PBS and iDC were fixed in 4% paraformaldehyde in PBS and put on slides. All slides were mounted with Prolong Gold antifade reagent containing Dapi (Invitrogen, Cat # P36941) covered with a glass coverslip, sealed, and stored in the dark at 4°C. Images were acquired with Zeiss LSM 800 AiryScan (z‐stack image) at 63x magnification. All images were analysed using Fiji software.

### 2.17 Cell Cycle Analysis

B16K1 cells were seeded in 6‐well dishes and cultured in complete medium. 24 h after, complete medium was replaced by serum free medium for 6 h. Then, cells were treated for 24 h with DDA at the indicated concentrations or the solvent vehicle (control). At the endpoint, the cells were detached with trypsin, cell and supernatant were centrifuged, washed, fixed in 70% ethanol/PBS and stored at ‐20°C overnight. Cells were incubated for 30 min at 37°C in the dark with a solution of propidium iodide (50 µg/mL), EDTA (2 mM) and RNase (DNAse‐free) (100 µg/mL) in PBS 1% BSA. Cell cycle analysis was performed by flow cytometry using MACSQuant VYB analyser (Miltenyi Biotec, France). Approximately 20 000 events were analysed from each samples. The percentages of cells within the Sub‐G1, GO/G1, S and G2/M phases of the cell cycle were calculated using FlowJo software (Ashland, Oregon, USA).

### 2.18 Cell Viability Assay

B16K1 cells and 4T1 cells were seeded in 6‐well dishes in the appropriated complete medium. After the indicated time, cells were treated with DDA at the indicated concentrations or the solvent vehicle (control) for 24 h. At the endpoint, cell death was determined using trypan blue exclusion assay. Briefly, cells were trypsinised, washed and suspended in PBS containing 0.4% trypan blue and incubated 5 min. Then, cells were counted in a Malassez cell under light microscopy. Cell viability was then quantified by counting viable (trypan blue‐negative) and dead cells (trypan blue‐positive).

### 2.19 Tumour Growth Analysis

C57BL/6 and BALB/c female mice (6‐ to 8‐week‐old) were purchased from Janvier Labs, France and maintained in specific pathogen‐free conditions. All of the animal procedures for the care and use of laboratory animals were conducted according to the guidelines of our institution and followed the general regulations governing animal experimentation (project: N°35503‐2022021814235803). Exponentially growing cells were harvested, washed twice in PBS and suspended in PBS. B16K1 cells (3 × 10^5^ cells diluted in 100 µL PBS) or 4T1 cells (5 × 10^4^ cells diluted in 100 µL PBS) were subcutaneously injected into the right flank of the syngeneic mice. Animals were treated intradermally in the opposite flank with 2 µg of the indicated sterile sEV, diluted in calcium free‐PBS, as indicated: either at day 0 and at day 7 or at days 7 and 10, after tumour establishment. For anti‐PD‐1 treatments, mice were injected intraperitonnally with anti‐PD‐1 antibody (Euromedex, France, Cat # BX‐BE0146, clone RMP1‐14) at 200 µg in 100 µL PBS per mouse, at day 13 and 15 after tumour cell injection, or with the isotype control anti‐IgG2a at the same concentration (Euromedex, France, Cat # BX‐BE0089, clone 2A3). Tumour volumes were measured every 2 or 3 days with a calliper and the volume was calculated using the formula [width^2^ × length]/2. Tumour weights were measured using a scale at the end of the experiments. The survival time of mice was recorded from the date of tumour inoculation to the date of death or sacrifice when the tumour volume reached 2,000 mm^3^.

### 2.20 Analysis by Flow Cytometry of T Cells Co‐cultured With sEV‐Pulsed DC

CD4+ and CD8+ T cells were isolated from C57B/L6 mouse spleens, cut into pieces and digested using the mouse Spleen Dissociation Kit (Miltenyi Biotec, France, Cat # 130‐095‐926) and then resuspended in RPMI 1640 medium. The tissues were mashed with an 18G syringe, after which the red blood cells were lysed (RBC lysis buffer, BioLegend, France, Cat # 420301). Then, cells were filtered through a 70 µm cell strainer and maintained at 4°C in FACS buffer. Total CD4+ T cells and naive CD8+ T cells were negatively selected and magnetically purified from single‐cell suspension of splenocytes using CD4+ T Cell Isolation Kit (Miltenyi Biotec, France, Cat # 130‐104‐454) or CD8a^+^ T Cell Isolation Kit (Miltenyi Biotec, France, Cat # 130‐104‐075) following the manufacturer's instruction.

Then, iDC that had been pulsed for 24 h with the indicated sEV or unpulsed iDC as control or LPS‐matured DC, were recovered and plated in 96‐well plates (0.1×10^6^ DC per well) and co‐cultured with CD4+ or CD8+ T cells (0.1×10^6^ T cells per well) in 200 µL of iDC culture medium supplemented with 2.5 µg of anti‐mouse CD3ε (eBioscience, Cat # 16‐0031‐86) and kept at 37°C for 48 h.

For intracellular cytokines production of DC‐activated T‐cells, cells were incubated with 3 µg/mL of BrefeldinA (Thermo Fisher Scientific, France, Cat # 00‐4506‐51) at 37°C for 4 h before endpoint. Then, cells were harvested by centrifugation at 400 x g for 5 min at 4°C and washed twice with PBS and pre‐incubated with Fc block antibody anti‐CD16/CD32 as described above. Samples were stained with LIVE/DEAD reactive dye, anti‐TCRβ (BD Bioscience, France, Cat # 563135, clone H57‐597, 1/50) and anti‐CD4 (BD Bioscience, France, Cat # 612952, clone GK1.5, 1/100) or anti‐CD8 (BioLegend, Netherlands, Cat # 100743, clone 53–6.7, 1/200) in FACS buffer for 30 min at 4°C. Then, cells were fixed and permeabilised using fixation/permeabilization staining buffer set (Thermo Fisher Scientific, France, Cat #00‐5523‐00) following the manufacturer´s instructions, washed with PBS and incubated overnight with anti‐T‐bet (Thermofisher Scientific, France, Cat # 505825–82, clone 4B10, 1/100) and anti‐ anti‐IFN‐γ (Miltenyi Biotec, France, Cat # 130‐117‐668, clone REA638,1/50) (Table S2) in FACS buffer at 4°C. Then, samples were washed twice with PBS. All samples were acquired using an LSRFortessa X‐20 (BD Biosciences), and data were analysed using FlowJo^TM^ v10 software (BD Life Sciences). Gating strategies are depicted in supplementary data files.

### 2.21 Isolation of Immune Cells From Tumours and Lymph Nodes

Tumours were removed on day 14, stripped of skin and fat, weighed, cut into pieces and digested using the mouse Tumour Dissociation Kit (Miltenyi Biotec, France, Cat # 130‐096‐730) and resuspended in RPMI 1640 medium. In addition, inguinal, cervical, axillary and brachial tumour draining lymph nodes from each mouse were pooled and digested with Dissociation Kit (Miltenyi Biotec, France, Cat # 130‐095‐926) then resuspended in RPMI 1640 medium. Tumours and lymph nodes were further mechanically disrupted with an 18G syringe and were depleted of red blood cells using an RBC lysis buffer (BioLegend, France, Cat # 420301) and then filtered through a 70‐µm cell strainer. Cells were washed in PBS, counted and maintained at 4°C with FACS buffer before proceeding with antibody‐mediated staining and analysed by flow cytometry as described below.

### 2.22 Analysis of Immune Cell Accumulation Into Tumours

For the analysis of the tumour immune infiltrate and myeloid cells in tumour‐draining lymph nodes, single‐cell suspensions were pre‐incubated for 20 min at 4 °C with Fc block antibody anti‐CD16/CD32 (Cat # 14‐0161‐85, clone 93, 1:200) in FACS buffer. For surface markers staining, the cells were stained with LIVE/DEAD reactive dye (Invitrogen, France, Cat # L34966A, 1:300) and with antibodies against the protein of interest: anti‐CD45 (BD Bioscience, France, Cat # 564279, clone 30‐F11, 1/200), anti‐TCRβ (BD Bioscience, France, Cat # 563135, clone H57‐597, 1:75), anti‐CD4 (BD Bioscience, France, Cat # 612952, clone GK1.5, 1/100), anti‐CD8 (BioLegend, Netherlands, Cat # 100743, clone 53–6.7, 1/200), anti‐CD11c (Thermo Fisher Scientific, France, Cat # 25‐0114‐82, clone N418, 1/200), anti‐CD80 (Thermo Fisher Scientific, France, Cat # 17‐0801‐82, clone B7‐1, 1/200), anti‐CD86 (Miltenyi Biotec, France, 130‐122‐129, clone REA1190, 1/200), anti‐MHC‐II (Miltenyi Biotec, France, Cat # 130‐112‐388, clone REA813, 1/200), anti‐CD11b (Miltenyi Biotec, France, 130‐113‐810, clone REA592, 1/200), anti‐CD197 (CCR7) (Thermo Fisher Scientific, France, Cat # 47‐1971‐82, clone 4B12, 1/100); anti‐CD24 (Miltenyi Biotec, France, 130‐110‐693, clone REA743, 1/100) and anti‐CD172a (Thermo Fisher Scientific, France, Cat # 46‐1721‐82, clone P84, 1/100) or controls isotypes, in FACS buffer for 30 min at 4°C. After surface markers staining, the cell suspensions were fixed and permeabilised using fixation/permeabilization staining buffer set (Thermo Fisher Scientific, France, Cat #00‐5523‐00) according to the manufacturer's instructions and then stained for intracellular targets: anti‐Ki‐67 (BD Bioscience, France, Cat # 556026 / 51–36524X, clone B56, 1:10), anti‐granzyme B (Miltenyi Biotec, France, Cat # 130‐120‐703, clone REA226, 1/50), anti‐ anti‐IFN‐γ (Miltenyi Biotec, France, 130‐117‐668, clone REA638, 1/50), anti‐Foxp3 (Thermo Fisher Scientific, France, Cat # 12‐4771‐82, clone NRRF‐30, 1/100). All samples were acquired using an LSRFortessa X‐20 (BD Biosciences), and data were analysed using FlowJo^TM^ v10 software (BD Life Sciences). All gating strategies are depicted in supplementary data files.

### 2.23 Lipidomic Analysis

For quantitative lipidomics, internal standards were added prior to lipid extraction. Cell homogenates or sEV were subjected to lipid extraction according to the protocol of Bligh and Dyer (Bligh and Dyer 1959). Lipids analysis was performed by direct flow injection analysis (FIA) using a triple quadrupole mass spectrometer (FIA‐MS/MS) and a high‐resolution hybrid quadrupole‐Orbitrap mass spectrometer (FIA‐FTMS). FIA‐MS/MS was performed in positive ion mode using the analytical setup and strategy described previously (Liebisch et al. 2004). FIA‐FTMS was performed as previously described (Höring et al. 2021). Diglycerides (DG) were recorded in positive ion mode *m/z* 500–1000 as [M+NH_4_]^+^ at a target resolution of 140,000 (at 200 *m/z*). PC were analysed in negative ion mode *m/z* 520–960 as [M+HCOO^−^] at the same resolution setting. BMP, lysophosphatidylglycerol (LPG), phosphatidylglycerol (PG) and phosphatidic acid (PA) were quantified by tandem mass spectrometry coupled to hydrophilic interaction chromatography (HILIC‐MS/MS) as previously described (Scherer et al. 2010). Briefly, lipids were extracted by buanolic extraction in the presence of internal standards. HILIC is used to separate isomeric BMP and PG. Raw data are listed in Table S4.

### 2.24 Statistical Analysis

Statistical analyses were carried out as specified in the figure legends using Graph Pad Prism software (v10). Statistical difference was considered when *P* < 0.05.

## 3 Results

### 3.1 BMP‐enriched DDA‐sEV Display Anti‐tumour Immune Response and Extend Mice Survival

**FIGURE 1:**
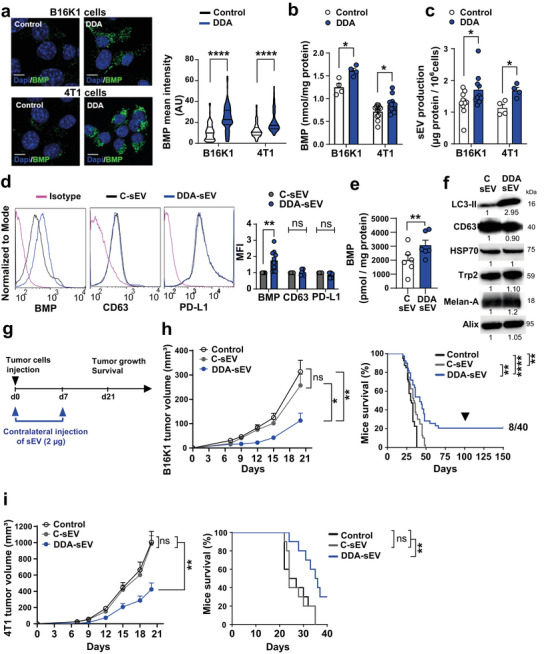
BMP‐enriched DDA‐sEV inhibit the growth of melanoma and TNBC cells and induce adaptive immunity. Both tumor cells treated for 24 h with either the solvent vehicle (control) or 2 µM DDA (melanoma B16K1) or 1 µM DDA (TNBC 4T1) and the sEV recovered after these treatments were analyzed as indicated. a) Representative confocal images of BMP immunostaining (green labelling) in the indicated tumor cells. Nuclear staining was performed with Dapi, scale bars, 10 nm. Violin plots indicate the quantification of mean intensity for BMP per cell, n = 4 independent experiments. b) Quantification of BMP by HILIC/MS/MS analysis in B16K1 (n = 4) and 4T1 cells (n = 12), treated or not with DDA. c) C‐sEV and DDA‐sEV recovered from B16K1 (n = 10) or 4T1 cells (n = 4), were analyzed by measuring protein content. d) Representative flow cytometry analysis and quantification of BMP, CD63 and PD‐L1 levels in C‐sEV and DDA‐sEV isolated from B16K1 cells treated as in a) (n = 5). e) Quantification of BMP by HILIC/MS/MS analysis of C‐sEV and DDA‐sEV isolated from B16K1 cells (n = 4). f) Representative immunoblot analysis of the indicated protein on C‐sEV and DDA‐sEV isolated from B16K1 cells treated with the solvent vehicle (control) or DDA as in (a), (n = 4), each lane was loaded with equal amounts of protein. Densitometry values indicate changes in protein expression relative to control treated cells and normalized to Alix. g) In vivo scheme of sEV treatment. Mice were implanted s.c. with B16K1 cells (h) or 4T1 cells (i) in the right flank. Then they were treated with either the solvent vehicle (control) or 2 µg of C‐sEV or DDA‐sEV, injected in the contralateral flank at day 0 and day 7. h‐i, left graphs) Mice (n = 20 mice/group) bearing B16K1 (h) or 4T1 (i) tumors were treated with PBS (control), 2 µg of C‐sEV or DDA‐sEV isolated from B16K1 or 4T1 cells. Mean tumor volumes (± SEM) of two independent experiments are shown, two‐way ANOVA and post‐test Tukey *P < 0.05, **P < 0.01, ns: not significant. h‐i, right graphs) Kaplan‐Meier curve analysis. Black arrow indicates the day of tumor re‐challenge with new parental tumor cells, numbers indicate the number of mice with total tumor regression out of the total number of mice. Log rank test (Mantel‐Cox) *P < 0.05, **P < 0.01, **P < 0.001, ****P < 0.0001, ns: not significant. a‐e) Data represent the mean ± SEM of the indicated experiments, Mann‐Whitney test *P < 0.05, **P < 0.01, ****P < 0.0001.

We previously reported that DDA increases BMP levels in B16F10 and SKMEL28 melanoma as well as E0771 breast adenocarcinoma cells and enhances the secretion of BMP‐enriched DDA‐sEV compared to C‐sEV isolated from solvent vehicle‐treated tumour cells (Record et al. 2022). To extend these findings, we analysed BMP accumulation in DDA‐sEV isolated from the media of mouse B16K1 melanoma and 4T1‐triple negative breast cancer (TNBC) cells. We also investigated the ability of DDA‐sEV to induce an anti‐tumour immune response in mice which had not been assessed in our previous work. Using immunofluorescence (IF) and liquid chromatography/mass spectrometry (HILIC‐MSMS), we showed that DDA, at concentrations that did not induce cell death but caused cell cycle arrest (Figure S1a‐d), significantly increased BMP immunostaining using the well‐characterised and specific anti‐BMP antibody (6C4) (Kobayashi et al. 1998) (Figure 1 a) as well as BMP levels (Figure 1 b). In addition, DDA increased DDA‐sEV secretion in both cell lines compared to C‐sEV (Figure 1 c). Analysis of sEV content showed that DDA‐sEV isolated from B16K1 (Figure 1 d‐f and Figure S2a) and 4T1 (Figure S1e‐f and Figure S2b) cells contained higher levels of BMP and LC3‐II, a marker of DDA activity, but exhibited no differences in the exosomal markers CD63, PD‐L1, Alix, or HSP70. Moreover, DDA‐sEV isolated from B16K1 and 4T1 were not enriched in the melanocytic antigens Melan‐A or Trp2 (Figure 1 f and Figure S2c) nor in the mammary antigen MFGE‐8 compared to C‐sEV respectively (Figure S1f and Figure S2d). In addition, tyrosinase was not detected in B16K1 cells, C‐sEV or DDA‐sEV isolated from these cells consistent with the fact that these cells do not produce melanin (not shown). These data indicate that DDA‐sEV isolated from these different cell lines share common exosomal markers and an enrichment in BMP. Next, we evaluated the anti‐tumour activity of DDA‐sEV on the growth of B16K1 or 4T1 tumours implanted into syngeneic immunocompetent mice, compared to the solvent vehicle (control) and C‐sEV treatments (Figure 1 g). Injection of DDA‐sEV secreted from DDA‐treated tumour cells significantly inhibited the growth of B16K1 (Figure 1 h and Figure S2e) and 4T1 tumours (Figure 1 i and Figure S2f) by 75 % and 60 % respectively compared to C‐sEV or solvent vehicle (control). In addition, DDA‐sEV treatment significantly prolonged survival of mice engrafted with B16K1 cells by 20 % at day 100 (Figure 1 h) and of mice engrafted with 4T1 cells by 30 % at day 40 (Figure 1 i), whereas no benefit was observed in control groups. Because DDA‐sEV‐treated mice displayed prolonged survival in the B16K1 model, they were re‐challenged with parental B16K1 tumour cells at day 100 (Figure 1 h, arrow). No tumour development was observed in the re‐challenged mice, indicating that they were vaccinated. These data indicate that DDA‐sEV isolated from melanoma or TNBC cells display antitumour activity, extend mouse survival and may induce a long‐term protective anti‐tumour immune response.

### 3.2 DDA‐sEV Promote Immune Cell Infiltration Into Tumours Implanted in Mice

**FIGURE 2:**
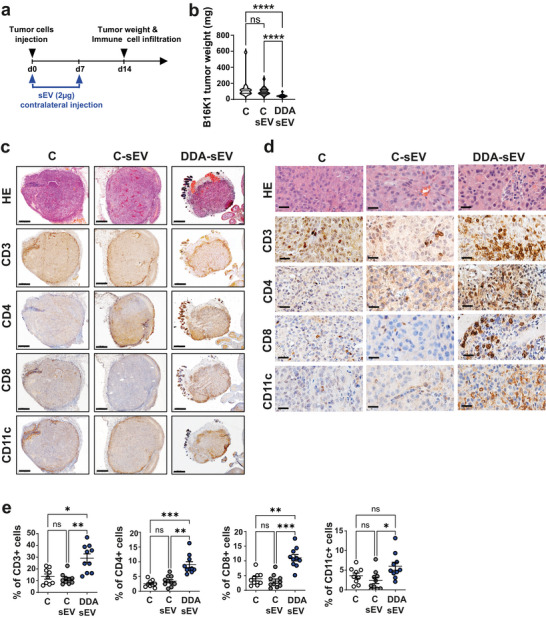
DDA‐sEV increase immune cell infiltration into tumors analyzed by IHC. a) In vivo scheme of sEV treatment and analysis. Mice were implanted with B16K1 tumor cells in the right flank and treated intradermally with 2 µg of the indicated sEV or the solvent vehicle (control), at day 0 and day 7, in the contralateral flank. Immune cell infiltration into tumor was analyzed at day 14. b) Mean tumor weight (± SEM) of B16K1 tumors harvested at day 14 is shown. Violin plots represent the mean of two independent experiments, (n = 14 mice/group), one‐way ANOVA and Kruskal Wallis post‐test ****P<0.0001, ns: not significant. c‐d) Representative pictures of tumors stained with either hematoxylin and eosin (HE) showing the decrease of tumor growth (scale bars: 500 nm) or stained with specific antibodies against, CD3+ (pan‐leukocytes), CD4+ and CD8+ T cells and CD11c+ myeloid cells (scale bars: 50 nm). e) Quantitative analysis of immune cells infiltrated into tumors treated as in c‐d and analyzed by IHC. Data represent the mean ± SEM; n = 10 mice/group (2 images analyzed per mice, using using Fiji software), one‐way ANOVA and Kruskal Wallis post‐test *P < 0.05, **P < 0.01, ***P < 0.001, ns: not significant.

**FIGURE 3:**
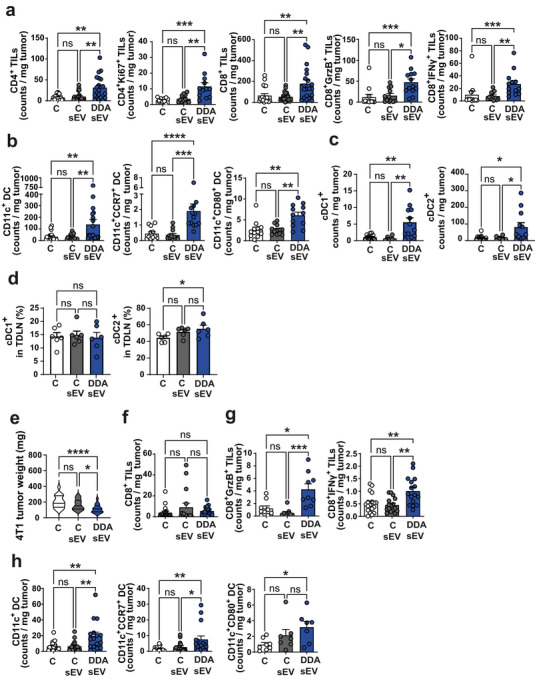
DDA‐sEV increase immune cell infiltration into tumors analyzed by flow cytometry. Mice were implanted with B16K1 cells (a‐d) or 4T1 cells (e‐h) and treated with sEV or the solvent control as described in Fig. 2a. Immune cell infiltration into tumors or tumor‐draining lymph nodes (TDLN) were analyzed on day 14. a‐d,e‐i) Flow cytometry analyses of the indicated immune cells accumulated into B16K1tumors (a‐c), d) into TDLN or 4T1 tumors (f‐h). Data represent the mean ± SEM of two independent experiments, (a,b) n = 14 mice/group), (c) cDC1+ were analyzed with the following markers: CD11c+ MHCII+ CD11b+ CD24+ CD172a‐ and cDC2+ were analyzed with the following markers: CD11c+ MHCII+ CD11b+ CD24‐ CD172a+, n = 12 mice/group, (d) n = 6 mice/group, e) 4T1 mean tumor weight (± SEM) is shown, violin plots represent the mean of two independent experiments, (n = 14 mice/group), the results were analysed for significance using one‐way ANOVA and the KruskalWallis post‐test. *P < 0.05, **P < 0.01, ***P < 0.001, ****P < 0.0001; ns: not significant.

We next analysed DC and T cell infiltration in B16K1 melanoma tumours grafted into mice treated with DDA‐sEV, C‐sEV, or solvent vehicle (control), as indicated (Figure 2a). On day 14, tumours were collected and weighed (Figure 2b) and tumour‐infiltrating immune cells were analysed (Figure 2c‐e). Tumours treated with DDA‐sEV were smaller than those in the control groups (Figure 2b‐c). Immunohistochemistry analyses of DDA‐sEV‐treated B16K1 tumours revealed a significant increase in CD3^+^, CD4^+^, CD8^+^ T cells, and CD11c^+^ myeloid cells within tumours compared to tumours treated with C‐sEV or solvent vehicle (Figure 2c‐e). Flow cytometry analyses showed that activated CD4^+^ and CD8^+^ T cells (Figure 3a) as well as mature DC (Figure 3b) were significantly increased in DDA‐sEV‐treated tumours compared to C‐sEV or solvent controls. We also assessed the accumulation of cDC1^+^ and cDC2^+^ subsets in tumours and tumour‐draining lymph nodes. DDA‐sEV significantly increased the accumulation of both cDC1 and cDC2 in tumours compared to controls, with cDC2 representing the predominant population (Figure 3c). In tumour‐draining lymph nodes, DDA‐sEV significantly increased cDC2 accumulation compared to control, but did not increase cDC1^+^ cells (Figure 3d).

Similarly, DDA‐sEV treatment significantly reduced the weight of 4T1 tumours compared to controls (Figure 3e) and was associated with increased accumulation of activated CD8^+^ T cells (Figure 3f‐g) and mature DC (Figure 3 h) into tumours, compared to control‐treated tumours. No infiltration of CD4^+^ or activated CD4^+^ infiltration into tumours was detectable (not shown). Together, these data indicated that in both models DDA‐sEV treatment activates an anti‐tumour immune response compared to C‐sEV or control conditions.

**FIGURE 4:**
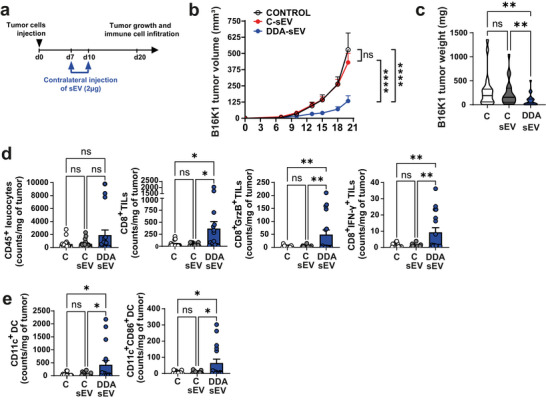
Treatment with DDA‐sEV activates anti‐tumor immune response in established tumors. a) In vivo sEV treatment scheme: On day 0, mice were subcutaneously implanted with B16K1 cells in their right flanks. On day 7, when tumors were measurable, the solvent vehicle (control), 2 µg of C‐sEV, or 2 µg of DDA‐sEV was injected intradermal into the opposite flank and on day 10, treatments were repeated. On day 20, immune cell infiltration into tumors were analyzed. b) Mean tumor volumes (± SEM) of two independent experiments are shown (n = 20 mice/group), two‐way ANOVA and post‐test Tukey, ****P<0.0001, ns: not significant. c) Mean tumor weights (± SEM) are shown. Violin plots represent the mean of two independent experiments, (n = 20 mice/group), one‐way ANOVA and Kruskal Wallis post‐test **P<0.01, ns: not significant. d‐e) Quantification by flow cytometry of the indicated immune cell infiltrated into tumors (n = 17 mice/group). Data represent the mean ± SEM of two independent experiments, one‐way ANOVA and Tukey post‐test *P<0.05, **P<0.01, ns: not significant.

To determine whether the activity of DDA‐sEV on the anti‐tumour immune response was due to interference with tumour cell engraftment rather than immunity‐mediated regression, we performed experiments on established tumours. Tumour cells were implanted on day 0 on the right flank, and the different treatments were administered on the left flank on days 7 and 10, once the tumours were established (Figure 4a). As shown in Figure 4b‐c, DDA‐sEV significantly reduced tumour growth and weight compared with C‐sEV or solvent control, demonstrating that their antitumour effect does not result from interference with tumour engraftment. Moreover, DDA‐sEV treatment increased the accumulation of activated T cells and DC within tumours (Figure 4d‐e), further supporting an immune‐mediated mechanism.

### 3.3 The BMP/EPCR Complex Is Involved in DDA‐sEV Uptake by iDC and iDC Maturation

**FIGURE 5:**
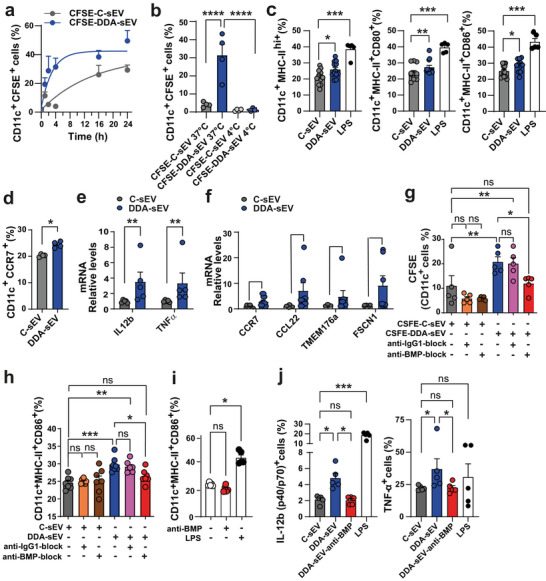
DDA‐sEV are more uptaken by DC and stimulate DC maturation. a‐b) Labelling of iDC with CFSE‐labelling sEV. CFSE‐labelling sEV (20 µg protein) were incubated with iDC for the indicated time either at 37°c for (a), (n = 6) or at 37°c or 4°c for 2h (b), (n = 4). The percent of cells labelled with CFSE‐labelling sEV was measured by flow cytometry. c) Expression of DC maturation markers analyzed by flow cytometry after treatment with 20 εg of C‐sEV or DDA‐sEV, (n = 12) or LPS used as a positive control (1 mg/ml), (n = 5). d) Expression of the migratory marker CCR7 analyzed in DC by flow cytometry after treatment with 20 εg of C‐sEV or DDA‐sEV, (n = 4). e‐f) mRNA levels of markers of functional mature DC analyzed by qPCR, (n = 5). g) The indicated CFSE‐labelled sEV, blocked with a specific anti‐BMP antibody or with a control isotype anti‐IgG1, or unblocked C‐sEV or DDA‐sEV were incubated with iDC for 2h and analyzed for their uptake by flow cytometry, (n = 5). h) sEV, blocked or not as in (g) were incubated for 24 h with iDC and then analyzed for maturation markers by flow cytometry, (n = 7). i) iDC were incubated with anti‐BMP antibody alone (1 µg/ml), the solvent vehicle (control) or LPS used as a positive control (1 mg/ml) for 24 h and then analyzed for maturation markers by flow cytometry (n = 5). j) iDC were incubated with 20 µg of C‐sEV or DDA‐sEV, or DDA‐sEV‐anti‐BMP or LPS used as a positive control (1 mg/ml) for 24 h and then analyzed for the intracellular expression of IL12b or TNFα proteins by flow cytometry, (n = 5). (a‐j) Data represent the mean ± SEM: (a) two‐way ANOVA and post‐test Tukey; (b‐c, e‐j) one‐way ANOVA and Kruskal Wallis post‐test, *P < 0.05, **P < 0.01, ***P < 0.001, ****P < 0.0001 and ns: not significant. (d) Mann‐Whitney test, *P < 0.05.

We next evaluated the uptake of DDA‐sEV or C‐sEV by mouse bone marrow‐derived immature DC (iDC) and their impact on iDC maturation. Similar amounts of DDA‐sEV and C‐sEV proteins (20 µg/sample) were incubated with carboxyfluorescein diacetate succinimidyl ester (CFSA‐SE). CFDA‐SE is a membrane‐permeable compound that becomes fluorescent only after cleavage by esterases present within the lumen of sEV, yielding CFSE, which then covalently binds primary amines inside the vesicles (Loconte et al. 2023). NTA confirmed that the CFSE‐C‐sEV and CFSE‐DDA‐sEV displayed comparable vesicles concentrations (1×10^1^ ^0^ particles/mL) and similar size (Figure S3a‐b). Flow cytometry analysis showed similar fluorescence intensities between CFSE‐C‐sEV and CFSE‐DDA‐sEV, indicating that CFSA‐DE is equally converted by esterases in both types of sEV (Figure S3c), confirming comparable dye conversion and incorporation. These controls validate that comparisons of their uptake by iDC are unbiased. Flow cytometry further revealed that the percentage of CFSE‐positive iDC increased more rapidly and to a higher extent when incubated at 37°C with CFSE‐DDA‐sEV compared with CFSE‐C‐sEV. The percentage of CFSE‐positive cells exposed to CFSE‐DDA‐sEV, increased up to approximately 4 h and then reached a stable plateau up to 24 h (Figure 5a). To distinguish true uptake from passive adsorption, we performed the same experiments at 4°C. The appearance of CFSE‐positive cells was completely abolished at 4°C for both CFSE‐C‐sEV and CFSE‐DDA‐sEV, indicating an energy‐dependent endocytic process rather than surface adsorption (Figure 5b). Immunofluorescence analyses further confirmed intracellular CFSE signals for both CFSE‐C‐sEV and CFSE‐DDA‐sEV in the form of punctate structures within iDC (Figure S3d‐f), suggesting that sEV are targeted within endosomal and/or endo‐lysosomal compartments (Joshi et al. 2020). Importantly, CFSE‐DDA‐sEV‐treated cells displayed significantly higher MFI than CFSE‐C‐sEV‐treated cells (Figure S3g). Together, these complementary approaches support the conclusion that DDA‐sEV are more efficiently internalised by iDC than C‐sEV, and that the CFSE signal does not result from sEV adhering to the cell surface.

Next, iDC were incubated for 24 h with DDA‐sEV or C‐sEV or LPS as a positive control and maturation markers were analysed. The cell surface expression of MHC‐II, CD80 and CD86, involved in DC maturation, was significantly increased in DC challenged with DDA‐sEV compared to DC challenged with C‐sEV as well as with LPS (Figure 5c). The cell surface expression of CCR7, a marker of DC migratory capacity, was also significantly increased with DDA‐sEV compared to C‐sEV (Figure 5d). This phenotypic maturation was accompanied by functional maturation, evidenced by significant increased mRNA expression of IL12b and TNFα (Figure 5e) as well as CCR7, CCL22, TMEM176A and FSCN1 (Figure 5f).

To study the role of BMP in DDA‐sEV uptake, CFSE‐labelled DDA‐sEV and CFSE‐labelled C‐sEV were incubated either with the well‐characterised anti‐BMP antibody (6C4) (Kobayashi et al. 1998) to block BMP (anti‐BMP‐block) or with an isotype control IgG1 (anti‐IGg1‐block). These sEV were then incubated with iDC and their uptake was analysed by flow cytometry. As shown in Figure 5 g, the uptake of CFSE‐C‐sEV blocked with anti‐BMP or anti‐IgG1 was similar to that of CFSE‐C‐sEV. In contrast, the uptake of CSFE‐DDA‐sEV blocked with anti‐BMP was significantly inhibited compared to DDA‐sEV, whereas blocking CSFE‐DDA‐sEV with an anti‐IgG1 did not affect their uptake by iDC. Next, DDA‐sEV or C‐sEV blocked with anti‐BMP or IgG1 were incubated with iDC to assess their ability to induce maturation. As shown in Figure 5 h, maturation marker expression was significantly increased in iDC incubated with DDA‐sEV or DDA‐sEV blocked with IgG1 compared to iDC incubated with C‐sEV. In contrast, DDA‐sEV blocked with anti‐BMP did not increase maturation marker expression compared to C‐sEV, DDA‐sEV or DDA‐sEV blocked with IgG1 (Figure 5 h). Treatment of iDC with anti‐BMP alone, used as a control, did not increase the expression of maturation markers compared to the control, unlike LPS which increased it significantly (Figure 5i). Furthermore, the intracellular accumulation of IL12b and TNFα proteins, representative of the functional maturation DC, was significantly increased in iDC incubated with DDA‐sEV, while no increase in the levels of these cytokines was observed in iDC incubated with anti‐BMP blocked DDA‐sEV or with C‐sEV, which showed equivalent levels (Figure 5j). LPS used as a control significantly increased IL12b intracellular accumulation but not that of TNFα (Figure 5j). These data indicate that BMP contributes to the functional maturation of DC induced by DDA‐sEV.

**FIGURE 6:**
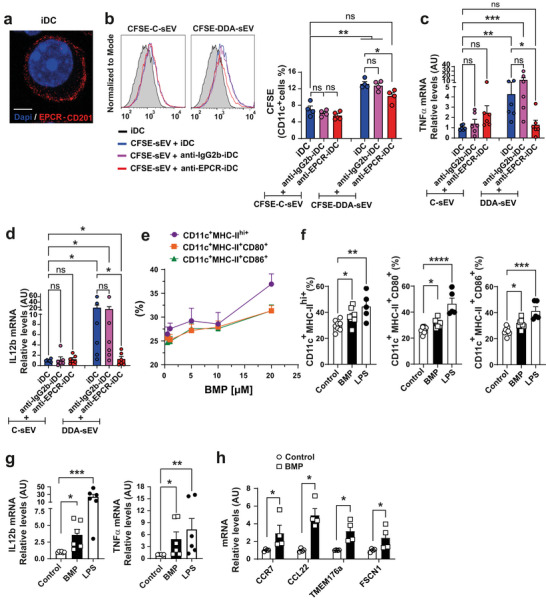
The BMP‐presenting receptor (EPCR) in iDC specifically uptakes DDA‐sEV that stimulate iDC maturation. a) Representative confocal microscopy image of iDC stained with anti‐EPCR‐CD201 (red). Nuclear staining was performed with DAPI. Scale bars: 10 εm (n = 3). b) Flow cytometry analysis of iDC, either blocked with anti‐IgG2b isotype or anti‐EPCR or not blocked with an antibody, iDC alone, incubated for 24 h at 37°C with CFSE‐labelled C‐sEV or DDA‐sEV by (n = 4). c‐d) TNF‐a (c) and IL12b (d) mRNA levels in mDC analyzed by qPCR that were treated as in Fig. 5b (n = 6). b‐d) Data represent the mean ± SEM, one‐way ANOVA and Kruskal Wallis post‐test *P < 0.05, **P < 0.01, ***P < 0.001, ns: not significant. e) iDC were treated for 24 h at 37°C with increasing concentrations of BMP and analyzed by flow cytometry for the expression of DC maturation markers. Data represent the mean ± SEM (n = 7). f) iDC were treated for 24 h with BMP (20 εM) n = 8, or LPS used as positive control (1 mg/ml) n = 5, or solvent vehicle (control) n = 8 and analyzed by flow cytometry for the expression of DC maturation markers. g‐h) iDC were treated for 24 h with BMP (20 εM) or LPS used as positive control (1 mg/ml), or solvent vehicle (control) and mRNA levels of the indicated functional DC maturation markers were quantified by qPCR, n = 6 for (g) and n = 4 for (h). f‐g) Data represent the mean ± SEM, one‐way ANOVA and Kruskal Wallis post‐test *P < 0.05, **P < 0.01, ***P < 0.001 and ****P < 0.0001. h) Data represent the mean ± SEM, Mann‐Whitney test *P < 0.05.

To confirm the importance of BMP in DDA‐sEV uptake and DC maturation, we analysed the effect of blocking of EPCR (CD201), which presents BMP (Müller‐Calleja et al. 2021), on DC. As shown in Figure 6a, iDC expressed EPCR at their cell surface. CFSE‐labelled DDA‐sEV and CFSE‐labelled C‐sEV were incubated with iDC pretreated with a specific anti‐EPCR blocking antibody or with an isotype anti‐IgG2b antibody as a control. The uptake of CFSE‐labelled C‐sEV into iDC or anti‐IgG2b‐blocked iDC or anti‐EPCR‐blocked‐iDC was similar (Figure 6b). In contrast, the uptake of CFSE‐labelled DDA‐sEV into anti‐EPCR‐blocked‐iDC was significantly inhibited compared with their uptake into unblocked iDC or anti‐IgG2b‐blocked‐iDC (Figure 6b). Consistent with these results, IL12b and TNF‐α expression showed the same pattern (Figure 6c‐d). These data showed that blocking the BMP‐presenting receptor EPCR in iDC inhibits DDA‐sEV uptake and functional DC maturation.

To confirm that BMP itself impacts DC maturation and that the observed effect on DC maturation with DDA‐sEV is not solely due to increased uptake of DDA‐sEV, we treated iDC with increasing concentrations of free BMP and assessed DC maturation. As shown in Figure 6 e‐f, incubation of iDC with increasing concentrations of BMP enhanced the expression of DC maturation markers MHC‐II, CD80 and CD86, similar to the effect observed with LPS, used as a positive control. This phenotypic DC maturation was accompanied by a functional maturation evidenced by increased mRNA expression of IL12b and TNFα (Figure 6 g) and of CCR7, CCL22, Tmem176a or Fscn1 (Figure 6 h). In contrast, similar experiments using increasing concentrations of free PC as a control did not induce DC maturation marker expression (Figure S3h). As control, we performed immunofluorescence analysis using the anti‐BMP antibody 6C4 on iDC treated with 20 µM free BMP or the vehicle control for 24 h. An increased 6C4 immunofluorescence signal was observed in BMP‐treated cells, whereas no increase was detected in control cells, indicating that BMP enters iDC (Figure S4). Together these data indicate that BMP *per se* stimulates functional DC maturation.

### 3.4 DDA‐sEV‐matured DC Activate CD4 T^+^ Cells and BMP Blockade in DDA‐sEV Inhibits This Process

**FIGURE 7:**
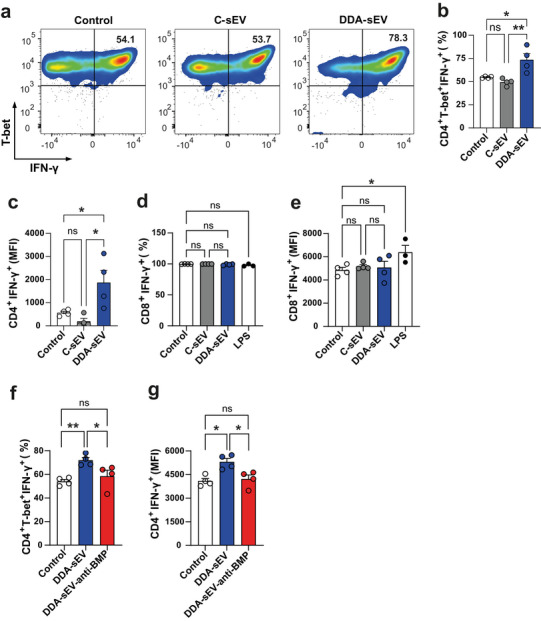
DDA‐sEV‐matured DC activate CD4 T+ cells and BMP blockade in DDA‐sEV inhibits this process. a‐b) Flow cytometry analysis of the reaction between DC matured with the indicated sEV and naïve CD4+ T cells. CD4+ T cells activated with DC incubated without sEV was used as control. a) Representative flow cytometry analysis. b) The mean percentage of CD4+T‐bet+ IFNγ+ activated by the control or the indicated sEV is shown (n = 4). c) The mean MFI of CD4+ INFγ+ is shown (n = 4). d‐e) Flow cytometry analysis of the reaction between DC matured with the indicated sEV or LPS (1 mg/ml) and naïve CD8+ T cells. CD8+ T cells activated with DC incubated without sEV was used as control. d) The mean percentage of CD8+IFNγ+ activated by the indicated sEV (n = 4) or LPS (n = 3) is shown (n = 4). e) The MFI of CD8+ INFγ+ activated by the indicated sEV (n = 4) or LPS (n = 3) is shown. f‐g) Flow cytometry analysis of the reaction between DC matured with the indicated sEV and naïve CD4+ T cells and analyzed as in (b‐c) respectively. b‐g) Data represent the mean ± SEM, one‐way ANOVA and Kruskal Wallis post‐test *P < 0.05, **P < 0.01, ns: not significant.

The quality of the functional maturation of iDC triggered by DDA‐sEV was assessed by analysing their ability to activate CD4^+^ or CD8^+^ T cells in mixed reaction assay compared to iDC treated with C‐sEV or untreated DC (control). DC matured by DDA‐sEV significantly increased the mean percentage of CD4^+^T‐bet^+^IFNγ^+^ T cells (Figure 7a‐b) and the mean fluorescence intensity (MFI) of IFNγ (Figure 7c), compared to DC stimulated with C‐sEV, for which both the percentage of CD4^+^T‐bet^+^IFNγ^+^ cells and the MFI of IFNγ were comparable to control (basal condition). In contrast, similar experiments performed with CD8^+^ T cells did not enhance the percentage of CD8^+^ IFNγ^+^ T‐cells (Figure 7d) or the mean MFI of IFNγ (Figure 7e) following DDA‐sEV, C‐sEV or control treatments, whereas LPS, used as a positive control, significantly increased the IFNγ MFI (Figure 7e) but not the percentage of CD8^+^ IFNγ^+^ T‐cells (Figure 7d). To investigate the role of BMP in DDA‐sEV‐induced Th1 CD4^+^ T cell response, similar experiments were performed with DDA‐sEV blocked with anti‐BMP. As shown in Figure 7f‐g, iDC incubated with DDA‐sEV‐anti‐BMP did not activate CD4+ T cell unlike iDC incubated with DDA‐sEV. Together these experiments revealed that DDA‐sEV‐matured DC enhances Th1 CD4^+^ T cell response and that BMP blockade in DDA‐sEV inhibits this process.

### 3.5 The DDA/LXRβ Complex Activates the Enzymes Involved in BMP Biogenesis in Tumour Cells

**FIGURE 8:**
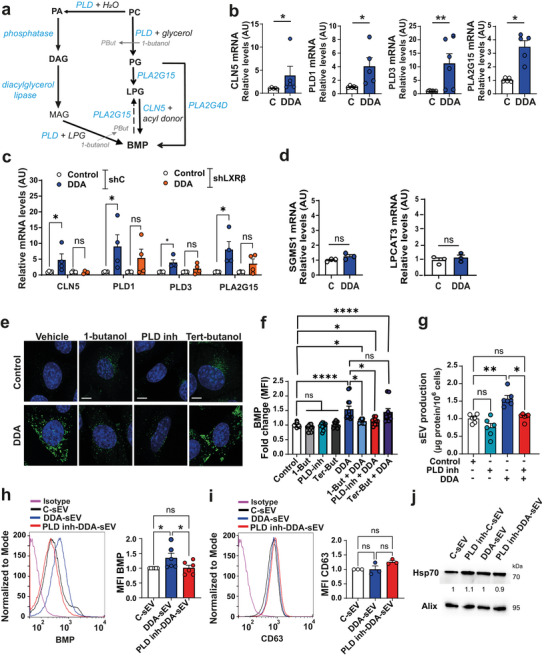
The DDA/LXRß complex upregulates the enzymes involved in BMP biogenesis. Inhibition of PLD1 impairs DDA‐induced BMP levels in cells and DDA‐sEV as well as DDA‐sEV production and function. a) Schematic diagram of the BMP biosynthetic pathway. b) Analysis by qPCR of the mRNA levels of the enzymes involved in BMP biogenesis, after 6h treatment of B16K1 cells with the solvent vehicle (control) or 2 µM DDA, (n = 5). Data represent the mean ± SEM (n = 5), c) Analysis by qPCR of mRNA levels of the enzymes involved in BMP biogenesis after 6h treatment of human SKMEL‐28 cells proficient (ShC) or deficient for LXRß (ShLXRß) with the vehicle (control) or 2 µM DDA, (n = 4). d) Analysis by qPCR of mRNA levels of the enzymes SGMS1 and LPCAT3 non‐related to BMP biogenesis (n = 3). b‐d) Data represent the mean ± SEM, Mann‐Whitney test *P < 0.05, **P < 0.01, ns: non significant. e‐j) B16K1 cells were treated for 24 h as indicated in the figures with the solvent vehicle (Vehicle) or 0.5 µM 1‐butanol or 10 nM VU0359595 (PLD1 inhibitor) or 0.5 µM Ter‐butanol in combination or not with 2 µM DDA. e) Representative confocal microscopy images of BMP immunostaining (green labelling) of B16K1 cells treated with the indicated compound (n = 4). Nuclei were stained with dapi (blue), scale bars: 10 nm. f) Quantification by flow cytometry of BMP levels on B16K1 cells treated with the indicated compound, (n = 5). g) sEV production recovered from 106 B16K1 cells treated with the indicated compound was analyzed by measuring protein content, (n = 6). h‐i) Representative flow cytometry quantification of markers of sEV (BMP (n = 6) and CD63 (n = 3), with densitometry values showing protein expression relative to C‐sEV and normalized to Alix (n = 3). (f‐i) Data represent the mean ± SEM, one‐way ANOVA and Kruskal Wallis post‐test, *P < 0.05, **P < 0.01, ***P < 0.001, ****P < 0.0001, ns: not significant.

During the course of this study, CLN5 was reported to be involved in BMP biogenesis through the transformation of lysophosphatidylglycerol (LPG) (Medoh et al. 2023) (Figure 8a). Another study indicated that intracellular phosphatidylglycerol (PG) generates BMP via the action of members of the cytosolic phospholipase A2 group IV (PLA2G4) family such as PLA2G4D (Bulfon et al. 2024), (Figure 8a). In addition, lysosomal PLD3 and PLD4 have been shown to synthesise BMP from monoacylglycerol (MAG) + LPG through their transphosphatidylation activity (Singh et al. 2024), (Figure 8a). Moreover, the phospholipase PLA2G15 has been reported to hydrolyse PG into LPG (Chen et al. 2023) and to hydrolyse some BMP species into LPG, except for the sn2, sn2’ BMP S,S isomer, which is resistant to hydrolysis (Nyame et al. 2025) (Figure 8a). It is well established that PLDs, particularly PLD1 and PLD2, hydrolyse PC into phosphatidic acid (PA) (Yao et al. 2021), which can then be converted into diacylglycerol (DAG) by dephosphorylation (Martin 1988). In addition, PLD may also contribute to the generation of PG from PC through a transphosphatidylation reaction (Zheng et al. 2003), (Figure 8a). PLD transphosphatidylation reaction allows incorporation of primary alcohols up to 4 carbon atoms in length into choline‐containing phospholipids by releasing the choline moiety from the PC molecule to form phosphatidylalcohol. Therefore, PLD transphosphatidylation can also exchange choline in PC with glycerol (primary alcohol of three carbon), which is present in biological fluids and culture media and can be imported into cells by aquaglyceroporine 3 to form PG. Thus, PLDs could act at two levels: generating PG from PC and generating BMP (S,S) from MAG and LPG through their transphosphatidylation activity (Singh et al. 2024) (Figure 8a). However, in the presence of a non‐physiological primary alcohol such as 1‐butanol, PLDs generate the inactive compound phosphatidylbutanol (PBut), which competes with the formation of PG or further downstream with that of BMP (Figure 8a).

Since the DDA/LXRβ complex increases BMP level in tumour cells (Record et al. 2022), we evaluated whether this complex activates the transcription of the enzymes described to be involved in BMP biosynthesis or hydrolysis (Figure 8a). As shown in Figure 8b, DDA significantly increased mRNA levels of CLN5, PLD1, PLD3 and PLA2G15 in B16K1 cells but not those of PLD2, PLD4 and PLA2G4D which are not expressed in this cell line (not shown). To determine the involvement of LXRβ on these effects, we used SKMEL28 cells proficient (shC) or SKMEL28 cells deficient for LXRβ (shLXRβ) expression (Segala et al. 2017, Record et al. 2022). A significant increase in mRNA levels of CLN5, PLD1, PLD3 and PLA2G15 was observed in shC SKMEL28 cells (shC) treated with DDA, but not in shLXRβ SKMEL28 cells deficient for LXRβ expression (Figure 8c). In contrast, as in B16K1, PLD2, PLD4 and PLA2G4D were not expressed in these cells (not shown). As controls, we assessed the effect of DDA on the transcription of other lipid metabolism enzymes unrelated to BMP biosynthesis. As illustrated in Figure 8d, DDA did not enhance the transcription of sphingomyelin Synthase 1 (SGMS1) or lysophosphatidylcholine Acyltransferase 3 (LPCAT3) enzymes. Together, these results indicate that DDA, via the LXRβ, specifically activates transcription of the CLN5, PLD1, PLD3 and PLA2G15 genes. Consistent with these findings, canonical LXR binding motifs were identified in the human and mouse promoter regions of CLN5, PLD1, PLD3 and PLA2G15 genes, using TFinder web portal (Minniti et al. 2025), (Table S1). Interestingly, analysis of cancer transcriptomic databases, using TNMplot.com (Bartha and Győrffy 2021), revealed that mRNA transcripts of CLN5 and PLD1 are downregulated in melanoma and breast cancer compared to normal tissue (Figure S5a).

**FIGURE 9:**
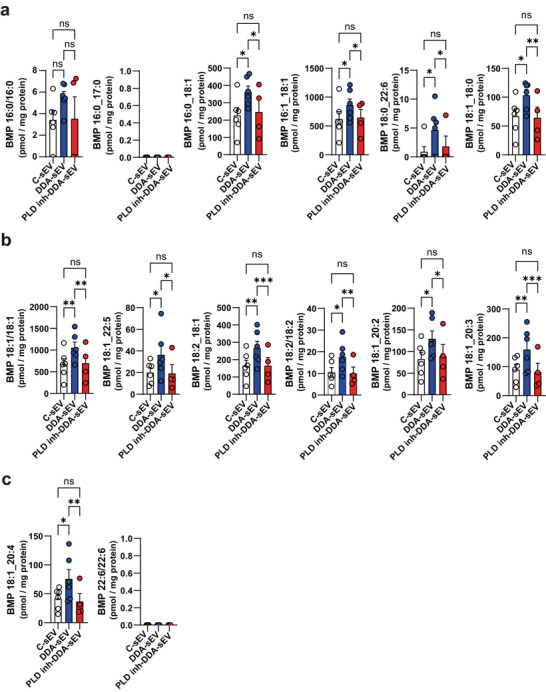
BMP enrichment in DDA‐sEV is inhibited by PLD1 inhibition in tumor cells. a‐c) C‐sEV, DDA‐sEV and PLD inh‐DDA‐sEV were isolated from B16K1 cells treated for 24 h with the solvent vehicle (control) or 2 µM DDA or 2 µM DDA + 10 nM VU0359595 (PLD1 inhibitor). Lipids were extracted and BMP levels were quantified by targeted lipidomic. Data represent the mean ± SEM, n = 6 for C‐sEV and DDA‐sEV and n = 4 for PLD inh‐DDA‐sEV, two‐way ANOVA and Tukey post‐test, *P < 0.05, **P < 0.01, ***P < 0.001, ns: not significant.

To investigate whether PLD contributes to DDA‐induced BMP synthesis, we interfered with PLD transphosphatidylation activity using either a primary alcohol (1‐butanol), which reacts with PC and blocks further reaction, or a PLD inhibitor (VU0359595) specific for PLD1. As expected, DDA‐treated cells exhibited a significant increase in BMP staining (Figure 8e) and BMP levels (Figure 8f) compared with control‐treated cells. Treatment with either 1‐butanol or the PLD1 inhibitor VU0359595 significantly reduced BMP staining and BMP levels in DDA‐treated cells (Figure 8e‐f). In contrast, tert‐butanol, a tertiary alcohol that cannot participate in the transphosphatidylation reaction, did not affect BMP staining or BMP levels in either control or DDA‐treated cells (Figure 8e‐f). These data indicate that DDA stimulates PLD‐dependent transphosphatidylation activity in tumour cells. Furthermore, treating tumour cells with DDA in combination with the PLD1 inhibitor significantly reduced sEV secretion (hereafter referred to as PLD inh‐DDA‐sEV) to levels comparable to C‐sEV (Figure 8 g) and significantly decreased BMP content in PLD inh‐DDA‐sEV (Figure 8 h), relative to DDA‐sEV. In contrast, the levels of other exosomal markers of sEV such as CD63, Hsp70 or Alix remained unchanged in DDA‐sEV or PLD inh‐DDA‐sEV compared to C‐sEV (Figure 8i‐j and Figure S5b). We then quantified BMP levels in DDA‐sEV compared to C‐sEV and PLD inh‐DDA‐sEV using targeted lipidomics. As shown in Figure 9a‐c, BMP levels, particularly 18:1 BMP species, were significantly higher in DDA‐sEV than in C‐sEV or PLD inh‐DDA‐sEV, thus confirming the BMP enrichment of DDA‐sEV compared to C‐sEV and the reduction in BMP levels in PLD inh‐DDA‐sEV.

**FIGURE 10:**
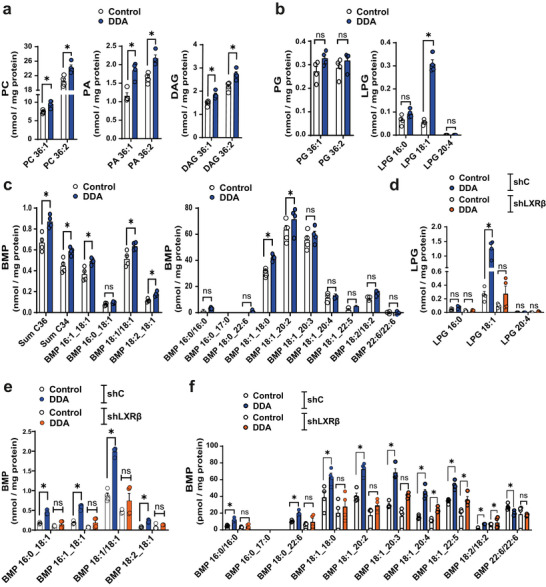
DDA stimulates the activity of the enzymes involved in BMP biogenesis. a‐f) Mouse B16K1 cells (a‐c) and human SKMEL‐28 cells proficient (ShC) or deficient for LXRß (ShLXRß) (d‐f) were treated for 24 h with the solvent vehicle (control) or 2 µM DDA. Lipids were extracted and quantitative targeted lipidomic was performed to analyze the substrates and products of the enzymes involved in BMP biogenesis. Data represent the mean ± SEM, n = 4, Mann‐Whitney test *P < 0.05, ns: not significant.

PLD1 and PLD2 are well known to catalyse the hydrolysis of PC to generate PA and choline (Yao et al. 2021) (Figure 8 a). Lipidomic analysis of B16K1 cells showed that DDA, compared to control treatment, significantly increased the PLD substrates PC 36:1 and PC 36:2, as well as the PLD products PA 36:1 and PA 36:2, indicating that DDA activates PLD hydrolase activity. DDA also increased the dephosphorylated products of PA, namely diacylglycerol (DAG) 36:1 and 36:2 (Figure 10 a and Figure 8 a), which can be converted into MAG and subsequently used to generate BMP via PLD transphosphatidylation activity (Singh et al. 2024). No increase in PG 36:1 and 36:2 was observed (Figure 10 b), likely due to its rapid conversion into LPG. Consistent with this, DDA significantly increased LPG 18:1 levels (Figure 10 b). Moreover, DDA significantly increased numerous 18:1‐containing BMP species (Figure 10 c). Both LPG (Figure 10 d) and BMP biosynthesis (Figure 10 e‐f) were significantly increased by DDA in shC‐SKMEL28 cells expressing LXRβ, whereas no increase was measured in shLXRβ SKMEL28 cells deficient for LXRβ expression. These findings are consistent with the fact that DDA stimulates, *via* LXRβ, the expression of the enzymes, PLA2G15, CLN5 and PLDs, involved in LPG and BMP production.

### 3.6 Reducing BMP Level in DDA‐sEV Inhibits Their Anti‐tumour Immune Function

**FIGURE 11:**
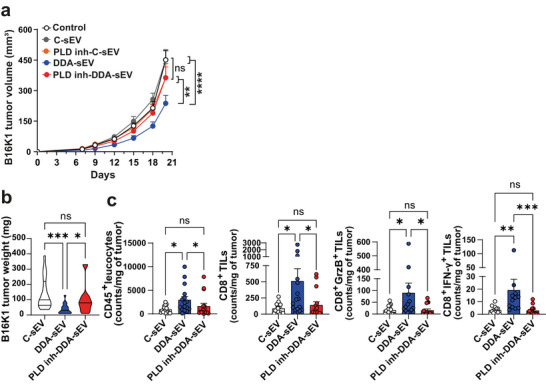
Reducing BMP level in DDA‐sEV, by treating tumor cells with a PLD inhibitor, abolished their anti‐tumor immune function. a) Mice bearing B16K1 tumors were treated, as in Fig. 2a, with the solvent vehicle (control) or 2 µg of the indicated sEV isolated from B16K1 cells treated with the solvent vehicle (Control) or 2 µM DDA (DDA‐sEV) or 2 µM DDA in combination with 10 nM of the PLD inhibitor VU0359595 (PLD inh‐DDA‐sEV). Mean tumor volumes ± SEM of two independent experiments are shown (n = 18 mice/group), two‐way ANOVA and Tukey post‐test, **P < 0.01, ****P < 0.0001, ns: not significant. b) On day 14, the tumors were removed and weighted, violin diagrams representing the mean of two independent experiments is shown (n = 16 mice/group). c) Flow cytometry analysis of the indicated immune cell infiltrating the tumor. Data represent the mean ± SEM of two independent experiments (n = 16 mice/group). b‐c) One‐way ANOVA and Kruskal Wallis post‐test, *P < 0.05, **P < 0.01, ***P < 0.001, ns: not significant.

We next evaluated the impact of reducing BMP levels on the anti‐tumour activity of DDA‐sEV by treating immunocompetent mice grafted with B16K1 cells with DDA‐sEV or PLD inh‐DDA‐sEV compared to controls. DDA‐sEV significantly reduced tumour growth, whereas PLD inh‐DDA‐sEV, C‐sEV or PLD inh‐C‐sEV did not elicit any antitumour effect (Figure 11a). Moreover, DDA‐sEV treatment significantly reduced tumour weight and increased the accumulation of activated CD8^+^ T cells within tumours compared to C‐sEV, whereas PLD inh‐DDA‐sEV failed to do so (Figure 11b‐c). These data confirm that BMP enrichment in DDA‐sEV is mediated by PLD‐dependent transphosphatidylation activity and is essential for DDA‐sEV anti‐tumour activity and immunogenic properties.

### 3.7 Blocking BMP on DDA‐sEV Prevents Their Anti‐tumour Immunogenicity

**FIGURE 12:**
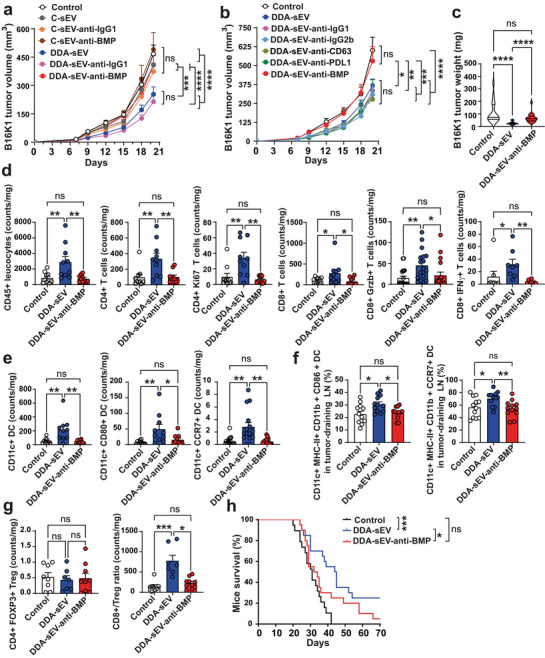
Blocking BMP on DDA‐sEV prevents their anti‐tumor immunogenicity. a) Mice (n = 18 mice/group) bearing B16K1 tumors were treated as in Fig. 1f with the solvent vehicle (control) or 2 µg of C‐sEV and DDA‐sEV isolated from B16K1 cells treated with the solvent vehicle or 2 µM DDA respectively, blocked or not with an anti‐IgG1 or anti‐BMP, mean tumor volumes are shown. b) Mice (n = 10 mice/group) bearing B16K1 tumors were treated as in Fig. 1f with the solvent vehicle (control) or 2 µg of sEV isolated from B16K1 cells treated with 2 µM DDA (DDA‐sEV) blocked or not with an anti‐IgG1 or anti‐IgG2b or anti‐BMP or anti‐CD63 or anti‐PD‐L1, mean tumor volumes are shown. c) Violin plots representing the mean ± SEM of B16K1 tumor weight at day 14, (n = 18 mice/group). d‐e,g) Quantification by flow cytometry of the indicated immune cell infiltrated into B16K1 tumors (n = 7 mice/group). f) Quantification by flow cytometry of the indicated immune cell infiltrated into the tumor draining lymph nodes (LN) (n = 12 mice/group). h) Kaplan‐Meier curve analysis (n = 20 mice/group). Log rank test (Mantel‐Cox) *P < 0.05, ***P < 0.001, ns: not significant. (a‐b) Data are the mean ± SEM of two independent experiments, two‐way ANOVA, *P < 0.05, **P < 0.01, ***P < 0.001, ****P < 0.0001, ns: not significant. (c‐g) Data represent the mean ± SEM, one‐way ANOVA and Kruskal Wallis post‐test *P < 0.05, **P < 0.01, ****P < 0.0001, ns: not significant.

We next studied the effect of BMP blockade on the properties of DDA‐sEV or C‐sEV isolated from B16K1 cells. DDA‐sEV and C‐sEV were incubated *in vitro* with the specific anti‐BMP antibody (6C4) to block BMP, or with an isotype control IgG1, washed to eliminate free antibodies, and subsequently injected into B16K1 cells‐grafted mice. BMP blockade on DDA‐sEV significantly inhibited their anti‐tumour activity to levels comparable to C‐sEV or C‐sEV blocked with anti‐BMP or IgG1, while blocking DDA‐sEV with IgG1 did not impair their anti‐tumour response (Figure 12a). To further confirm the specificity of BMP blockade, we incubated DDA‐sEV with antibodies against CD63 or PD‐L1, which recognise their extracellular epitopes along with their respective isotype controls. Blocking CD63 or PD‐L1 did not inhibit the anti‐tumour activity of DDA‐sEV, wheras BMP blockade with anti‐BMP significantly inhibited their anti‐tumour effect (Figure 12b). As controls, each antibody alone was injected at the same doses used for sEV blockade. None altered tumour growth (Fig S5c). We then assessed the impact of BMP blockade on DDA‐sEV‐mediated immune cell infiltration at day 14, as described in Figure 2a. DDA‐sEV significantly decreased tumour weight while DDA‐sEV‐anti‐BMP did not (Figure 12c). DDA‐sEV significantly increased the accumulation of activated CD4+ and CD8^+^ T cells (Figure 12d) and mature DC (Figure 12e) within tumours. DDA‐sEV also significantly increased mature DC in tumour‐draining lymph nodes, indicating that DDA‐sEV drive the expansion of mature DC in the tumour microenvironment and at peripheral sites (Figure 12f). In contrast, DDA‐sEV blocked with anti‐BMP failed to induce any of these effects (Figure 12d‐f). DDA‐sEV had no effect on CD4^+^Foxp3^+^ regulatory T cells (Treg) compared to controls or BMP‐blocked DDA‐sEV, but significantly increased the CD8^+^/Treg ratio (Figure 12 g). Moreover, the activation of anti‐tumour immunity induced by DDA‐sEV was associated with increased mice survival compared to control or DDA‐sEV‐anti‐BMP groups (Figure 12 h).

Similarly, we next evaluated BMP blockade on DDA‐sEV isolated from 4T1 cells using the anti‐BMP antibody (6C4). In the 4T1 tumour model, DDA‐sEV significantly reduced tumour weight whereas DDA‐sEV‐anti‐BMP did not (Figure S6a). DDA‐sEV also significantly increased the accumulation of activated CD8^+^ T cells within tumours, while BMP blockade on DDA‐sEV inhibited this effect (Figure S6b). These results indicate that BMP enrichment in DDA‐sEV is a critical determinant of their anti‐tumour immunogenicity in both melanoma and TNBC models.

### 3.8 DDA‐sEV Increase Anti‐PD‐1 Efficacy Against Melanoma Implanted Onto Mice

**FIGURE 13:**
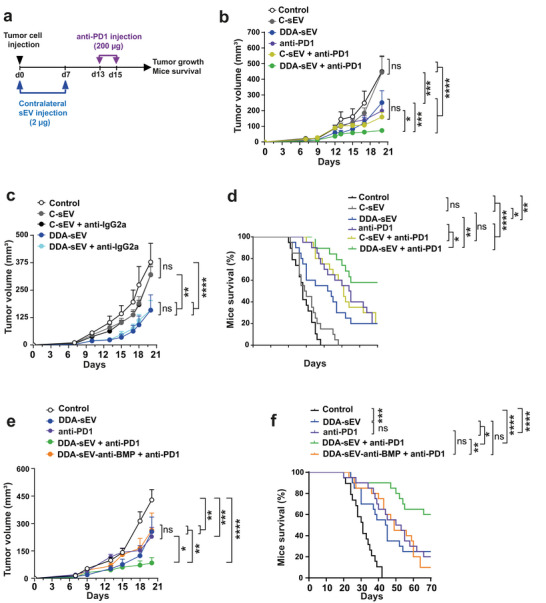
DDA‐sEV enhance anti‐PD 1 therapy. a) Schematic diagram of sEV and anti‐PD‐1 treatment in vivo. Mice implanted with B16K1 tumor cells were treated with sEV as in Fig.1f. At day 13 and 15, mice were treated (i.p.) with anti‐PD‐1 antibody (200 µg per mice) or the solvent vehicle (control). b) Mice (n= 18 mice/group) bearing B16K1 tumors were treated with the solvent vehicle (control), C‐sEV or DDA‐sEV with or without anti‐PD‐1 antibody, mean tumor volumes are shown. c) Mice (n = 9 mice/group) bearing B16K1 tumors were treated with C‐sEV or DDA‐sEV with or without anti‐IgG2a (isotype) (200 µg per mice) or the solvent vehicle (control), mean tumor volumes are shown. d) Kaplan‐Meier curves analysis, n = 20 mice per group. Log rank test (Mantel‐Cox) *P < 0.05, **P < 0.01, ****P < 0.0001, ns: not significant. e) Mice (n= 18 mice/group) bearing B16K1 tumors were treated with the solvent vehicle (control) or DDA‐sEV with or without anti‐PD‐1 antibody that was blocked or not with anti‐BMP antibody, mean tumor volumes are shown. f) Kaplan‐Meier curve analysis of the experience in (e) treated as in (d), n = 20 mice per group. Log rank test (Mantel‐Cox) *P < 0.05, ***P < 0.001, ns: not significant. (b,c,e) Data are the mean ± SEM of two independent experiments, two‐way ANOVA and Tukey post‐test, *P < 0.05, **P < 0.01, ***P < 0.001, ****P < 0.0001, ns: not significant.

Data from the literature indicate that tumour‐sEV are key contributors to resistance mechanisms against checkpoint inhibitor therapy in melanoma, primarily due to their immunosuppressive properties (Han et al. 2022, Chen et al. 2018, Poggio et al. 2019, Lyu et al. 2024). We therefore evaluated whether DDA‐sEV could enhance the therapeutic efficacy of anti‐PD‐1 in melanoma. Mice grafted with B16K1 melanoma cells were treated with DDA‐sEV or C‐sEV at day 0 and 7, followed by intraperitoneal administration of anti‐PD‐1 or IgG isotype, at day 13 and 15 (Figure 13a). DDA‐sEV and anti‐PD‐1 monotherapies each inhibited melanoma growth to a similar extend, while C‐sEV had no effect relative to control (Figure 13b). Pre‐treatment of mice with DDA‐sEV significantly enhanced the efficacy of anti‐PD‐1 therapy compared to DDA‐sEV alone, while C‐sEV did not influence the response to anti‐PD‐1 therapy (Figure 13b). Experiments performed with IgG2a isotype did not modify the effect of DDA‐sEV or C‐sEV (Figure 13c). Furthermore, DDA‐sEV or anti‐PD‐1 monotherapies significantly improved mouse survival at day 70 by 20% compared to controls, whereas combined DDA‐sEV + anti‐PD‐1 treatment increased survival to 58% (Figure 13d). No mice survived in the control or C‐sEV groups (Figure 13d).

We next investigated whether BMP enrichment in DDA‐sEV, known to enhance functional DC maturation, contributed to the improved anti‐PD‐1 response. DDA‐sEV were blocked with anti‐BMP antibody and tested in combination with anti‐PD‐1 therapy. BMP blockade on DDA‐sEV prevented the synergistic effect observed with DDA‐sEV + anti‐PD‐1, resulting in tumour growth inhibition comparable to anti‐PD‐1 monotherapy (Figure 13e and Figure S6c). Moreover, at day 70, mouse survival in the DDA‐sEV blocked with anti‐BMP + anti‐PD‐1 group was reduced to 10 % compared with 60 % survival in the DDA‐sEV + anti‐PD‐1 group (Figure 13f). Overall, these results highlight that BMP enrichment in DDA‐sEV is essential for their anti‐tumour immune activity and for their capacity to enhance anti‐PD‐1 therapy in melanoma.

## 4 Discussion

The present study highlights the importance of increasing BMP levels in tumour sEV to stimulate anti‐tumour immune response and to enhance the efficay to anti‐PD‐1 therapy in melanoma. This was realised by the DDA/LXRβ complex, which stimulates BMP biosynthesis in tumour cells and its enrichment in DDA‐sEV. Indeed, we show that DDA increases, in tumour cells, the expression of the enzymes CLN5 and PLDs, recently described involved in BMP biogenesis, and enhances their activity by elevating the levels of their products (LPG, PA, DAG, and BMP). We further confirm that PLDs, here PLD1 and/or PLD3, participate in BMP biosynthesis in tumour cells through their transphosphatidylation activity, as BMP increase in DDA‐treated cells and DDA‐sEV is blocked by 1‐butanol or a PLD1 inhibitor. Our previous work showed, using different methods, that BMP content in tumour cells and DDA‐sEV is increased by DDA acting on the LXRβ (Record et al. 2022). Here, we confirm this finding and show that the expression of PLD1, PLD3, CLN5 and PLA2G15, enzymes involved in the BMP biosynthetic pathway, is under the transcriptional control of the DDA/LXRβ complex. These results emphasizes the central role of LXRβ and its endogenous ligand DDA, in the pharmacological regulation of BMP biogenesis, opening new therapeutic opportunities in cancer and various lysosome‐associated diseases linked to impaired BMP biogenesis (Gruenberg 2020, Medoh et al. 2023, Singh et al. 2024).

Using multiple approaches, we demonstrate that BMP is an essential anti‐tumour immunogenic determinant in DDA‐sEV. BMP contributes *in vivo* to tumour growth inhibition, increased infiltration of activated CD8^+^ T cells, CD4^+^ T cells and DC in melanoma and TNBC models and enhanced uptake and functional maturation of iDC *ex vivo*, which increased Th1 response of CD4^+^ T cell. Consistent with these findings, treatment of iDC with exogenous free BMP promotes concentration‐dependent functional DC maturation, indicating that BMP acts, through a receptor‐mediated mechanism, beyond facilitating faster and increased sEV uptake. Supporting this model, we showed that DDA‐sEV uptake by iDC requires the interaction of BMP with EPCR (Müller‐Calleja et al. 2021), which is expressed on the surface of iDC. To date this specific BMP/EPCR‐dependent route of sEV uptake by iDC has not been previously described. Because BMP enrichment in DDA‐sEV secreted by DDA‐treated tumours promotes antitumour immune response, a decrease in BMP levels in cancer may contribute to immune suppression and tumour development, suggesting that pharmacological induction of BMP biosynthesis could have significant therapeutic benefit. In line with this hypothesis, RNAseq analyses revealed that CLN5 and PLD1 expression is decreased in melanoma and breast tumours compared to normal tissue (Figure S5a). Furthermore, our study shows that DDA‐sEV treatment enhances anti‐PD‐1 therapy and improves mouse survival and that BMP enrichment in DDA‐sEV is required for these improved responses in melanoma.

Immune Checkpoint Inhibitors (ICI) such as anti‐PD‐1 have shown significant efficacy in the treatment of metastatic melanoma (Larkin et al. 2019). However, more than 50% of patients fail to respond or relapse after treatment (Cortes et al. 2022) and no robust biomarker currently predict resistance or relapse. Our results suggest that BMP biosynthesis and the expression of BMP biosynthetic enzymes may serve as biomarkers of response to anti‐PD‐1 therapy. Supporting this idea, low CLN5 expression was significantly associated with non‐response to anti‐PD‐1 treatment in melanoma patients (Accession Numbers: GSE78220) (Hugo et al. 2016) and (Figure S6d).

In conclusion, this study highlights the crucial importance of BMP enrichment for the uptake of DDA‐sEV by iDC and their functional maturation which increased Th1 response of T cells, enabling the induction of antitumour immunogenicity through cytotoxic T cell activation and enhancing the efficacy of anti‐PD‐1 therapy. Our work identifies the BMP biosynthetic pathway involving PLD1, PLD3 and CLN5 as being under the pharmacological control of LXR*β* and DDA in tumour cells. Therefore, inducing BMP biogenesis with DDA may represent a highly promising strategy in cancer immunotherapy, and BMP levels in tumour and sEV, together with the expression of BMP biosynthetic enzymes in tumour, could serve as relevant biomarkers of response to anti‐PD‐1 therapy.

## Author Contributions

**Julio Buñay**: investigation, methodology, writing – review and editing, formal analysis, visualization, validation, data curation. **Michel Record**: funding acquisition, validation, visualization, writing – review and editing, formal analysis, investigation, conceptualization, methodology. **Philippe de Medina**: investigation, methodology, validation, visualization, writing – review and editing, formal analysis. **Silia Ayadi**: investigation, methodology, validation, writing – review and editing, formal analysis, visualization. **Laly Pucheu**: investigation, methodology, validation, visualization, formal analysis. **Céline Colacios**: investigation, methodology, validation, visualization. **Bruno Ségui**: investigation, validation, visualization, methodology. **Marcus Höring**: investigation, validation, visualization, methodology, formal analysis. **Gerhard Liebisch**: investigation, methodology, validation, visualization, writing – review and editing, formal analysis. **Hélène Martin**: methodology, formal analysis. **Marc Poirot**: conceptualization, investigation, funding acquisition, writing – original draft, methodology, validation, visualization, writing – review and editing, formal analysis, data curation, supervision. **Sandrine Silvente–poirot**: conceptualization, investigation, funding acquisition, writing – original draft, methodology, validation, visualization, writing – review and editing, formal analysis, supervision, data curation, project administration.

## Funding

This work was supported by recurrent grants from INSERM, CNRS and Toulouse University, the Institut National du Cancer (INCA) (grant PLBio 20–160) and La Ligue Contre Le Cancer (Equipe labellisée par la Ligue Nationale Contre le Cancer).

## Ethics Statement

All of the animal procedures for the care and use of laboratory animals were conducted according to the guidelines of our institutions and followed the general regulations governing animal experimentation.

## Consent

The authors have nothing to report.

## Conflicts of Interest

JB, MR, PDM, MP and SSP have filed a patent related to this work. The other authors have no competing interests to disclose.

## Permission to reproduce material from other sources

We did not reproduce material from other sources.

## Health and Safety

We confirm that all mandatory laboratory health and safety procedures have been complied with in the course of conducting all the experimental work reported in our paper.

## Manuscript approval by authors

All authors reviewed and approved this version of the manuscript.

## Supporting information




**Supplementary Figure**: jev270225‐sup‐0001‐Figures.docx

**Supplementary Table**: jev270225‐sup‐0002‐TableS1.xlsx

**Supplementary Table**: jev270225‐sup‐0003‐TableS2.xlsx

**Supplementary Table**: jev270225‐sup‐0004‐TableS3.xlsx

**Supplementary Table**: jev270225‐sup‐0005‐TableS4.xlsx

## Data Availability

The data for the study are available from the corresponding authors upon request.
