# Supplementary material for: The Phospholipid Bis(monoacylglycero)Phosphate Confers Antitumour Immunogenicity to Exosomes Secreted by Dendrogenin A, Which Activates Its Biosynthesis in Tumour Cells
Source: J Extracell Vesicles. 2026 Jan 30;15(2):e70225. doi: 10.1002/jev2.70225 (PMC12859387; doi:10.1002/jev2.70225)
Supplement: Supplementary file 1 — Supplementary Figure: jev270225‐sup‐0001‐Figures.docx [file JEV2-15-e70225-s002.docx]

**Supplementary figures and legends**

**
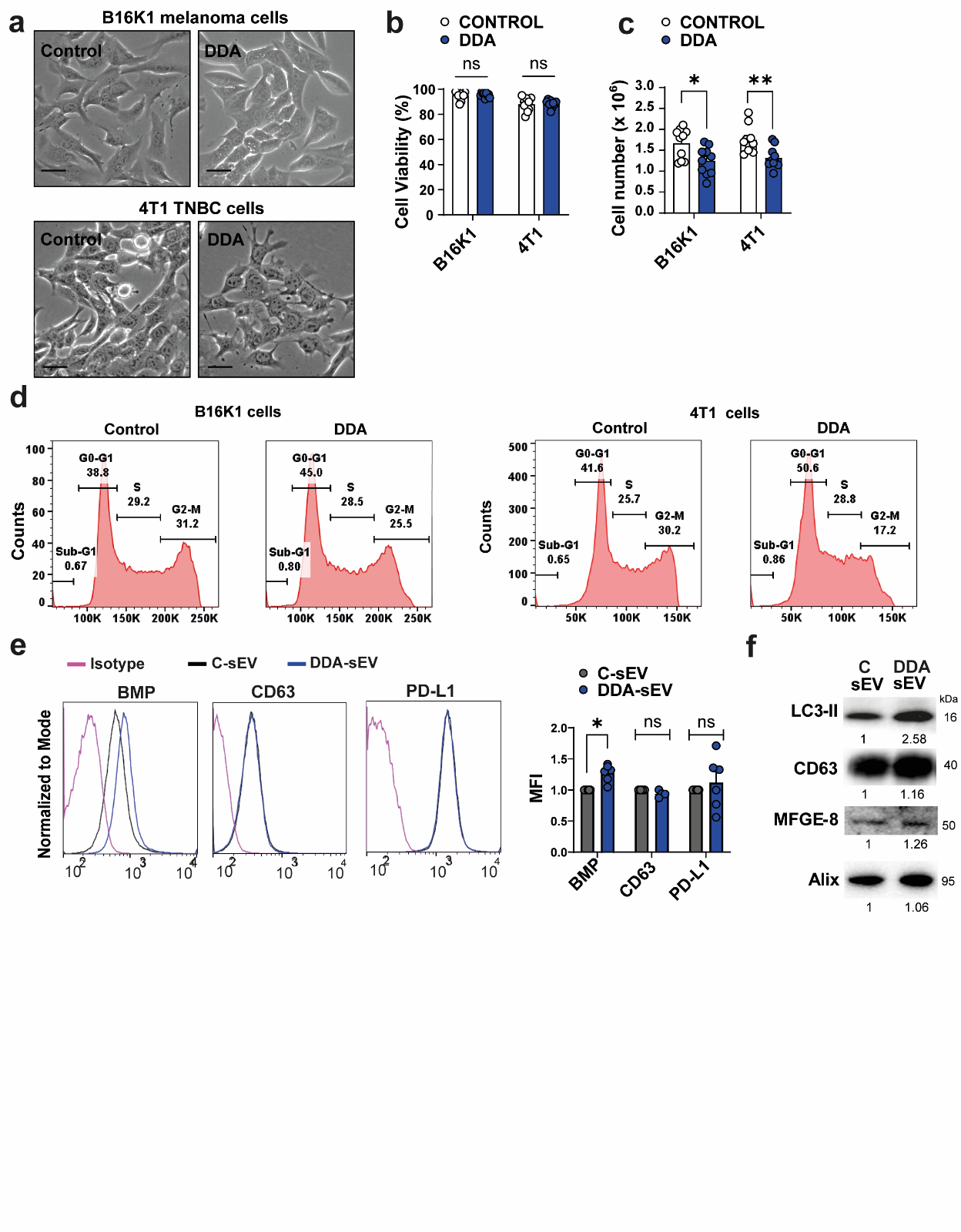
**

**Fig. S1. Impact of DDA on cell viability and proliferation in melanoma and TNBC models**

a-d) B16K1 melanoma cells and 4T1 TNBC were treated with the solvent vehicle (control) or DDA at 2 or 1μM respectively for 24 h. a) Representative images of cells by light microscopy (×40), (n=3). b-c) Quantification of cell viability and cell number by trypan blue assay. Data represent the mean ± SEM, n=10, Mann-Whitney test, *P<0.05, **P< 0.01, ns: not significant. d) Representative flow cytometry analysis of cell cycle distribution, percentages of cells in each cell cycle phase are indicated (n=3). e) Representative flow cytometry analyses of the content of C-sEV and DDA-sEV isolated from 4T1 cells analyzed as in Fig. 1d, data represent the mean ± SEM, n=4, Mann-Whitney test, *P<0.05, ns: not significant. f) Representative immunoblot analyses of the content of C-sEV and DDA-sEV isolated from 4T1 cells analyzed as in Fig. 1e.


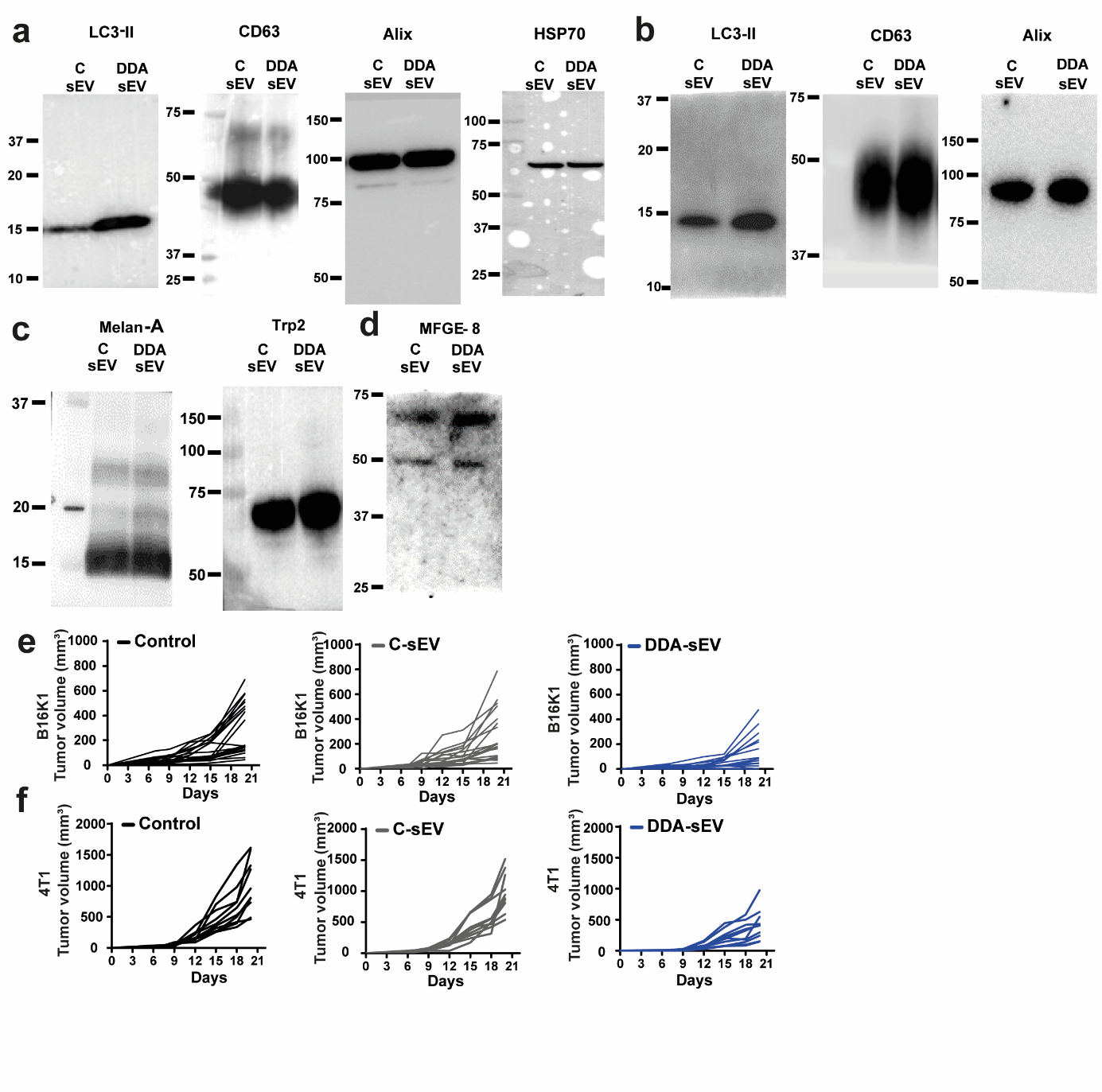


**Fig. S2. Expression by immunoblot analysis of the protein of interest in sEV isolated from B16K1 or 4T1 cells and B16K1 and 4T1 tumor growth in individual mouse**

a-d) Immunoblot analysis of the expression of the indicated proteins in C-sEV or DDA-sEV isolated from B16K1 cells (a-c) or 4T1 cells (b-d). e-f) B16K1 (a) and 4T1 (b) tumor growth in each individual mouse for each treatment of Fig.1g-h is shown.


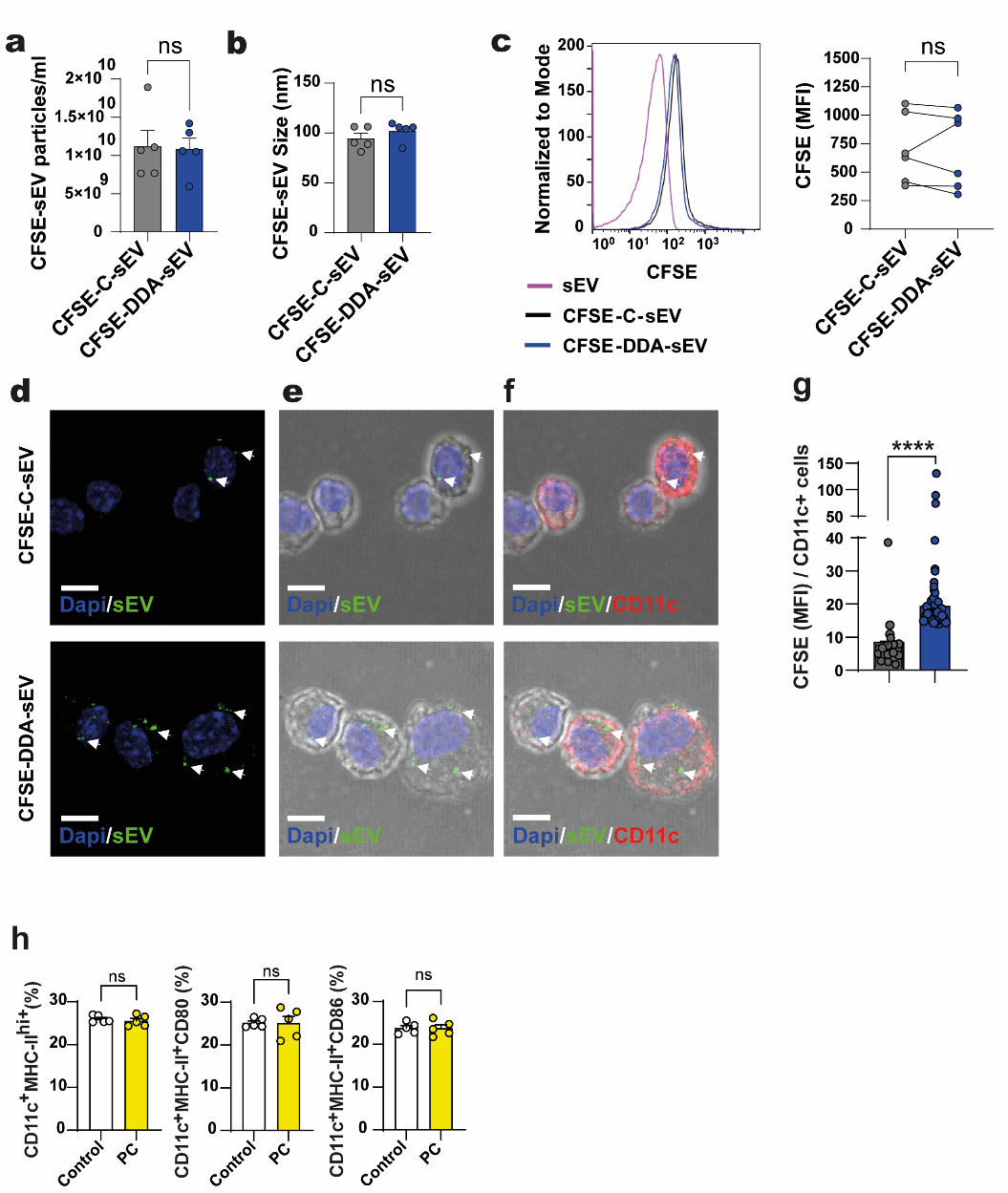


**Fig. S3. Analysis of sEV uptake by iDC**

a-c) Analysis of CFSE-sEV incubated with iDC. a) Analysis of the concentration of the particles present in the CFSE-sEV samples incubated with iDC using NTA indicating that the same concentrations of CFSE-C-sEV and CFSE-DDA-sEV were incubated with iDC (n=5). b) Analysis of the size of the indicated CFSE-sEV particles, using NTA and mode size values, (n=5). c) Representative flow cytometry analysis and quantification of CFSE fluorescence intensity of CFSE-C-sEV and CFSE-DDA-sEV incubated with iDC (n = 6), indicating similar labelling between C-sEV and DDA-sEV. (a-c) Data represent the mean ± SEM, Mann–Whitney test (ns: not significant). d-g) Fluorescence analysis of CFSE-C-sEV and CFES-DDA-sEV incubated for 24 h with iDC, showing the internalization of CFSE+ sEV into iDC (d-f). g) Quantitative analysis of the fluorescence uptaken by iDC in (d), data represent the mean ± SEM of MFI for CFSE on per positive DC (CD11c+), n=3 independent experiments. Mann–Whitney test, ***P<0.001. h) iDC were treated for 24 h with PC (20 µM) or the solvent vehicle (control) and analyzed by flow cytometry for the expression of DC maturation markers. Data represent the mean ± SEM (n = 5), Mann–Whitney test, ns: not significant.


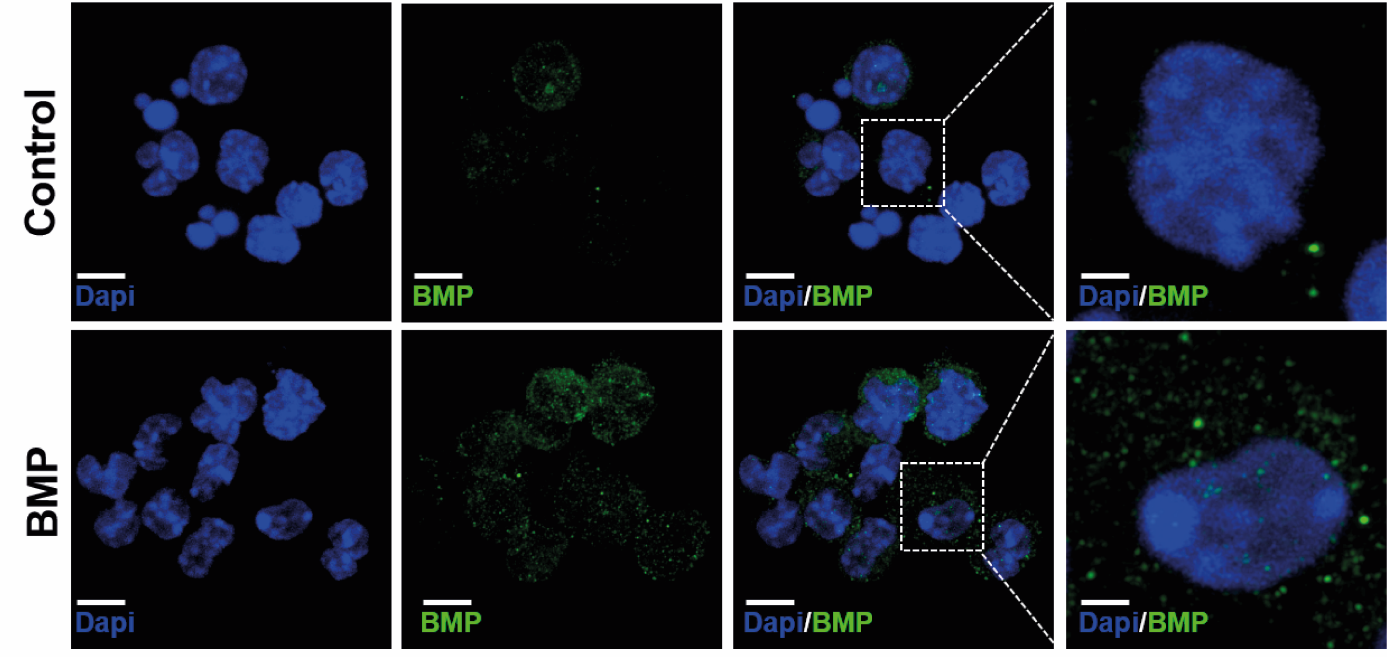


**Figure S4**. **immunofluorescence analysis shows that free BMP enters iDC**

iDC were treated for 24 h with 20 µM BMP or the vehicle control and analyzed by immunofluorescence with the anti-BMP antibody 6C4.


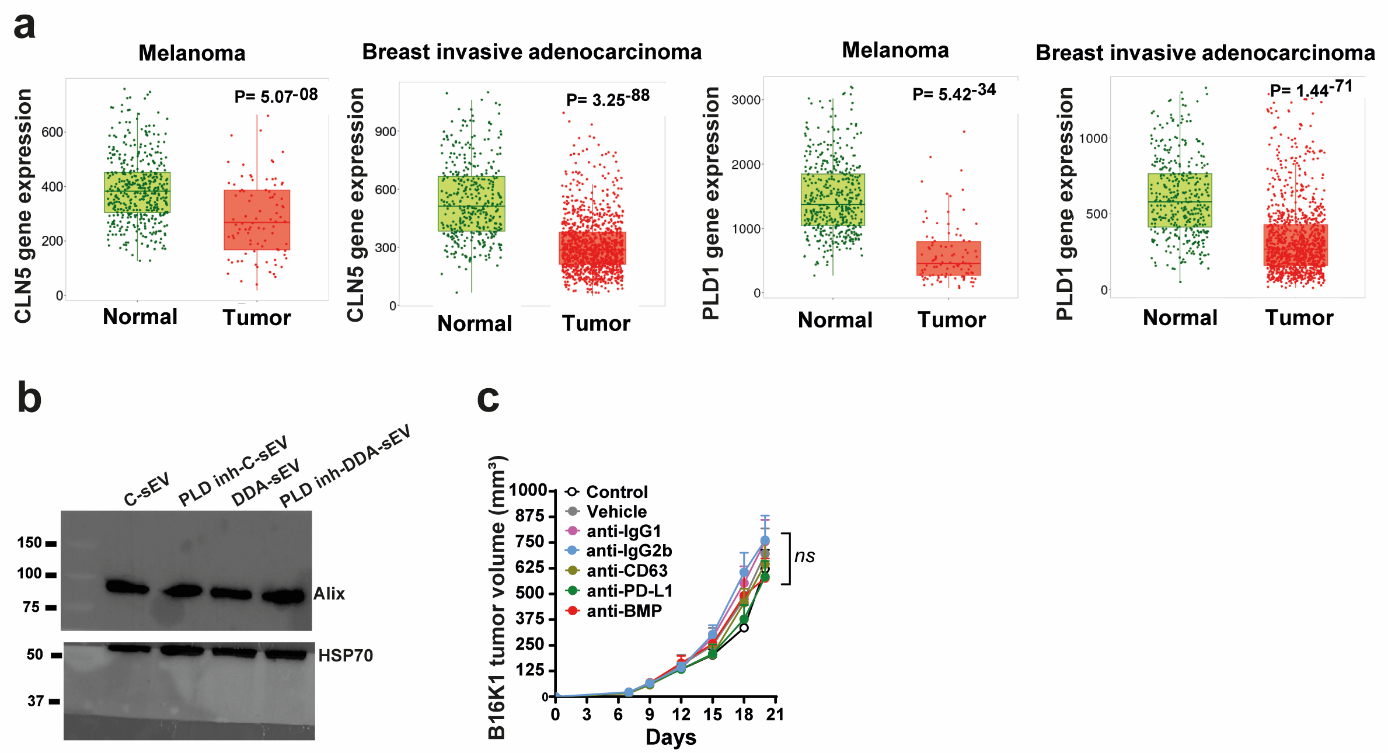


**Figure S5.RNASeq analysis of CLN5 and PLD1 gene expression in the indicated tumor and normal tissue**

a) RNASeq analysis of CLN5 and PLD1 gene expression in melanoma (n=103) compared to normal adjacent tissue (n=474) and in breast invasive carcinoma (n=403) compared to normal adjacent tissue (n=1097) using TNMplot.com (47). b) Immunoblot analysis of the expression of the indicated protein in sEV isolated from B16K1 cells treated with the solvent vehicle (C-sEV) or DDA (DDA-sEV) in presence or not of the PLD1 inhibitor. c) Mice (n =5 mice/group) bearing B16K1 tumors were treated in the contralateral flank on day 0 and day 7 with the solvent vehicle (control) or each antibody used in (Fig. 12a-b) at concentrations used for sEV blockade. Mean tumor volumes ± SEM are shown, two-way ANOVA, ns: not significant.


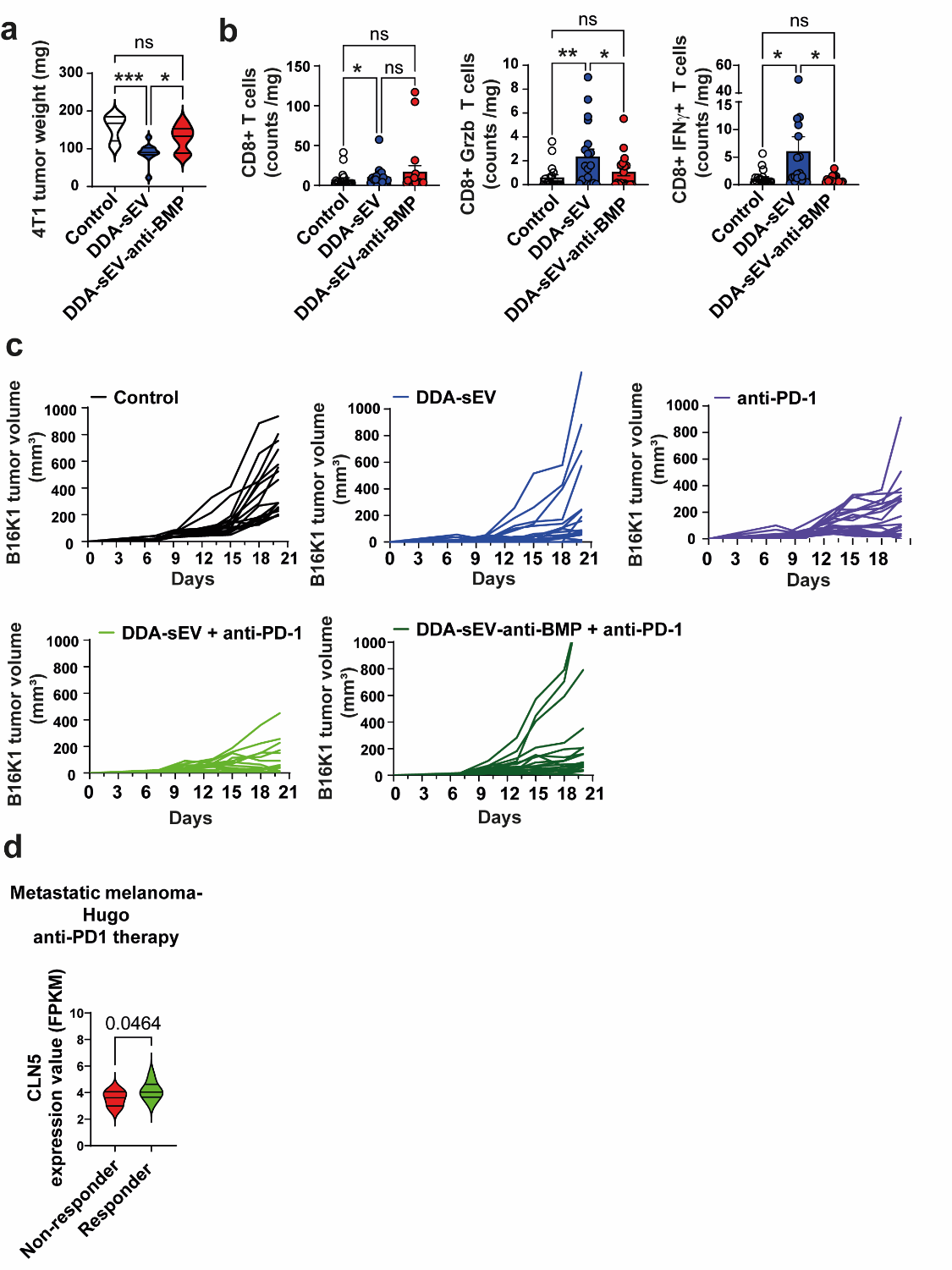


**Figure S6. DDA-sEV anti-tumor immune response in 4T1 cells requires BMP enrichment**

a-b) Mice were implanted with 4T1 tumor cells in the right flanck and treated two times at day 0 and 7 in the contralateral flanck with the solvent vehicle or 2 µg of C-sEV or DDA-sEV as in Fig. 1f. Tumors were analyzed at day 14. a) Mean tumor weight (± SEM) was analyzed (n=10 mice/group). Violin plots represent the mean of two independent experiments, one-way ANOVA and Kruskal Wallis post-test *P< 0.05, ***P<0.001, ns: not significant. b) Flow cytometry analyses of the indicated immune cells infiltrated into 4T1 tumors. Data represent the mean ± SEM of two independent experiments (n = 19 mice/group), one-way ANOVA and Kruskal Wallis post-test *P< 0.05, **P<0.01, ns: not significant. c) Individual B16K1 tumor growth of each individual mouse for each treatment of Fig. 8e-f is shown. d) Violin plots of CLN5 gene expression in melanoma samples of Hugo-cohorts (Vanderbilt Cancer-Immune data portal) of patients treated with anti-PD-1 therapy. Unpaired t test with Welch's correction, *p<0.05.


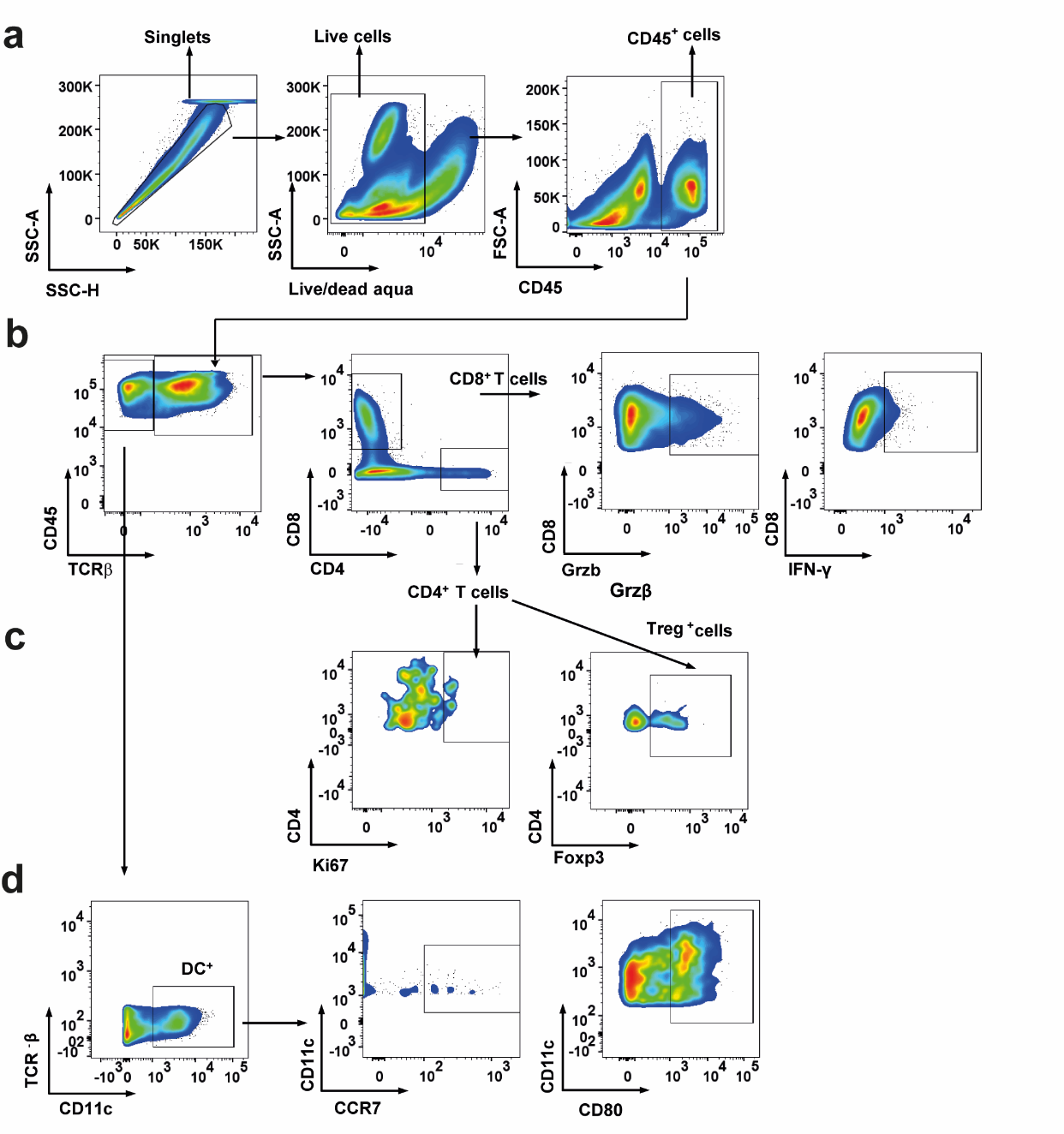


**Fig S7. Tumor infiltrate analysis of CD4^+^ and CD8^+^ T cells and mature DC**

a-d) Flow cytometry gating strategy to identify tumor-infiltrating CD4^+^ and CD8^+^ T cells and mature DC.


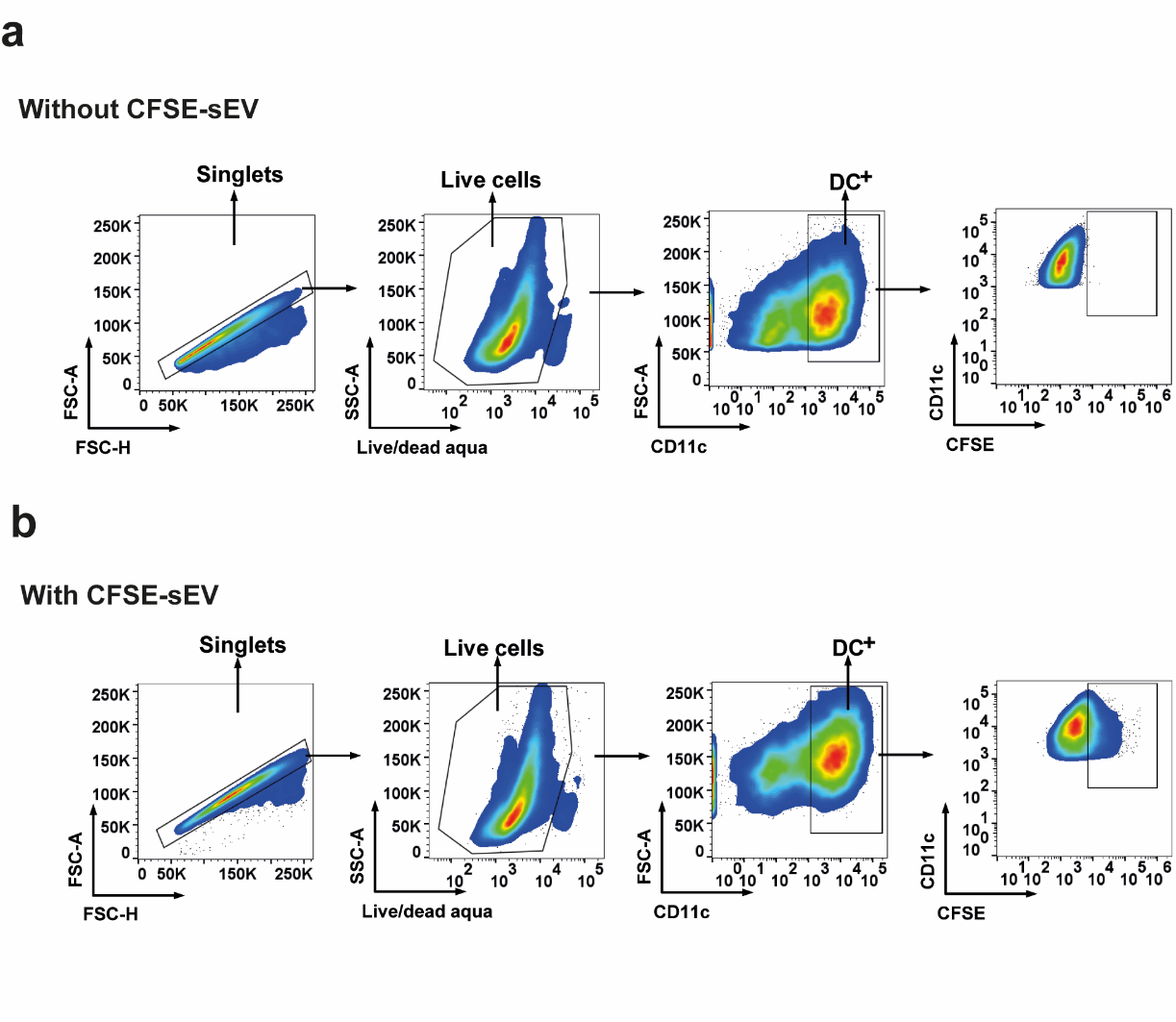


**Fig S8. CFSE-sEV uptake analysis**

a-b) Flow cytometry gating strategy to analyse CFSE-sEV uptake by iDC.


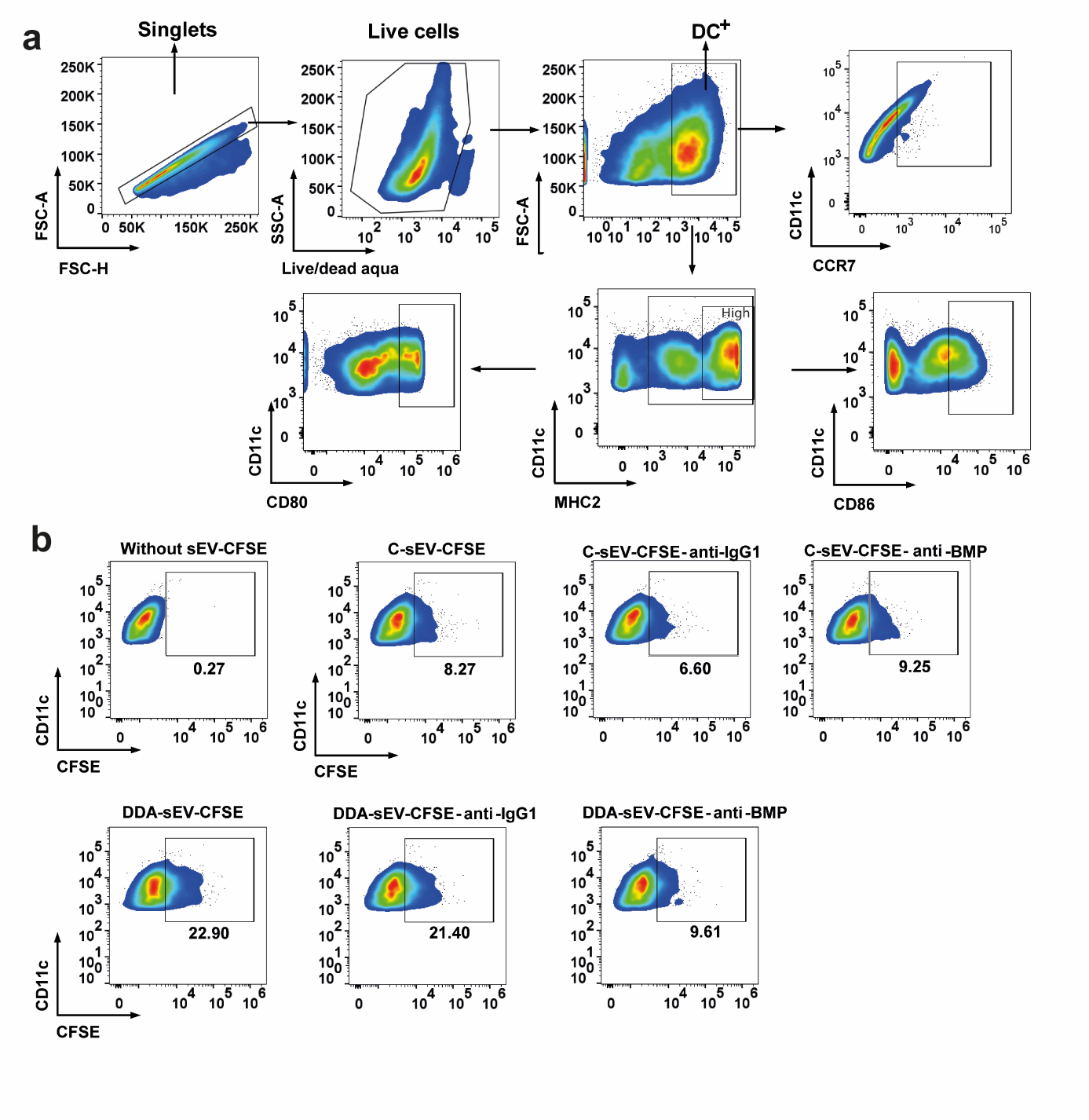


**Fig S9. Analyses of DC maturation by sEV**

a) Flow cytometry gating strategies to identify mature DC. b) Flow cytometry scatter plots showing the percentage uptake of CFSE-labelled C-sEV or DDA-sEV by iDC.


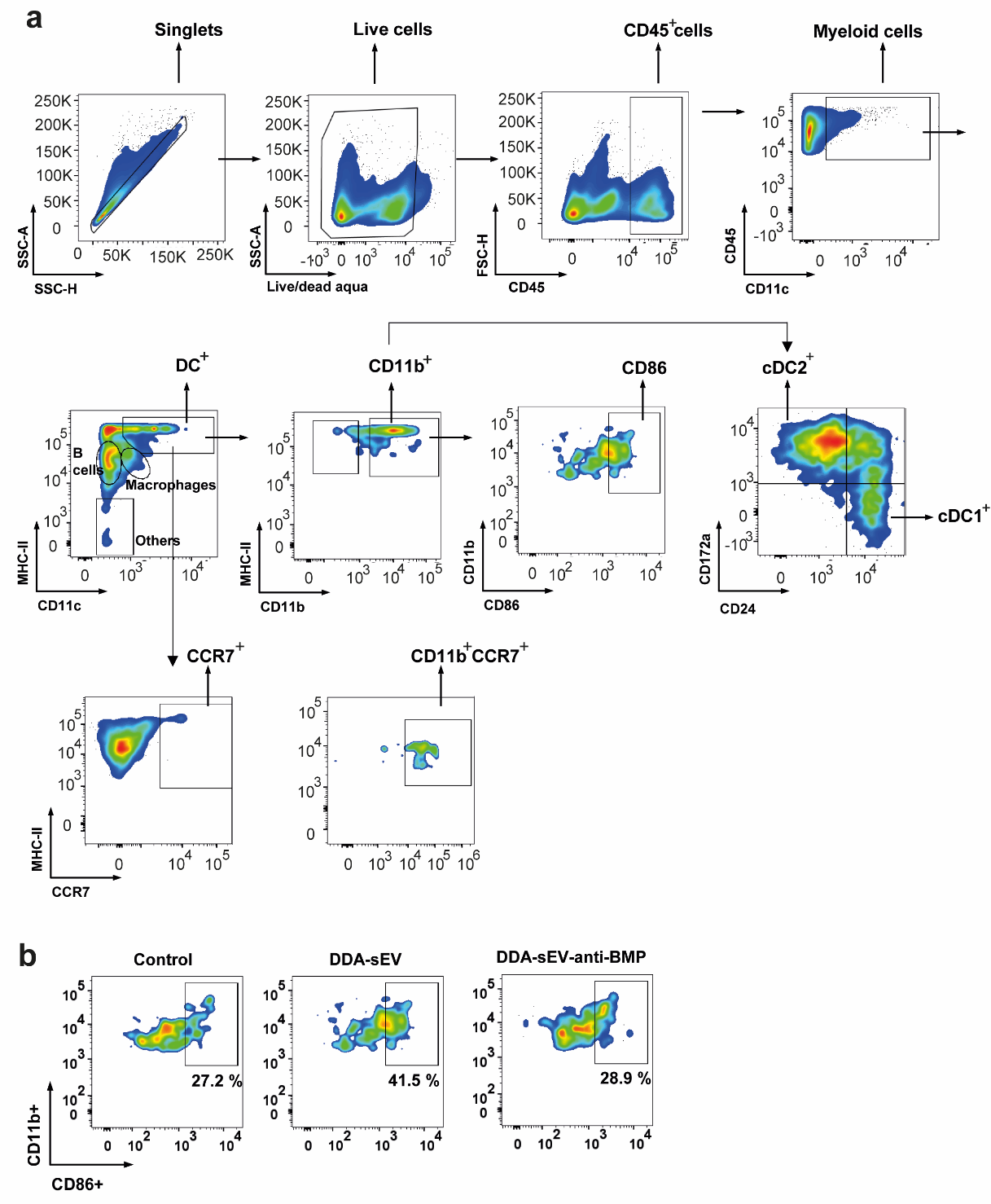


**Fig S10.** **Analysis of DC in draining lymph nodes**

a) Flow cytometry gating strategies to identify mDC in lymph nodes. b) Flow cytometry scatter plots showing the percentage of mDC in lymph nodes after treatment with the solvent vehicle (control), DDA-sEV or DDA-sEV blocked with anti-BMP antibody.


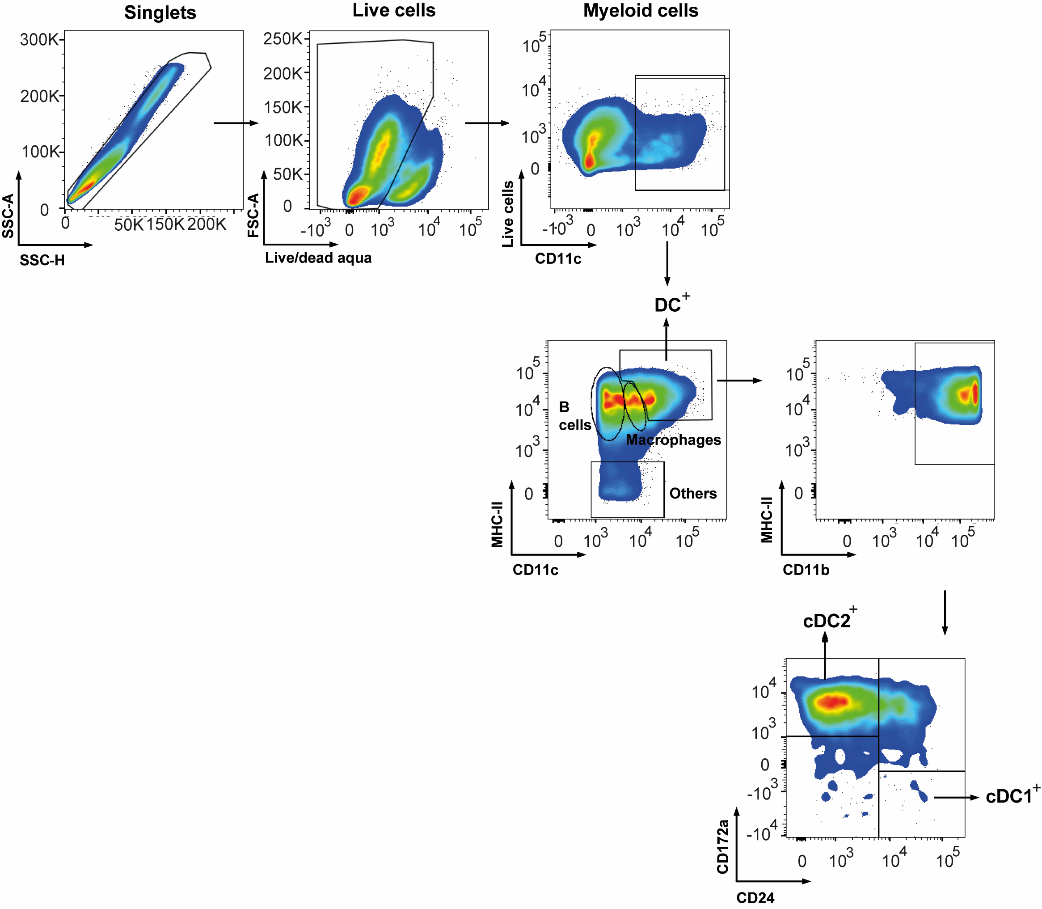


**Fig S11.** **Analysis of cDC1 and cDC2 in tumors**

Flow cytometry gating strategies to identify cDC1 and 2 in tumor

**
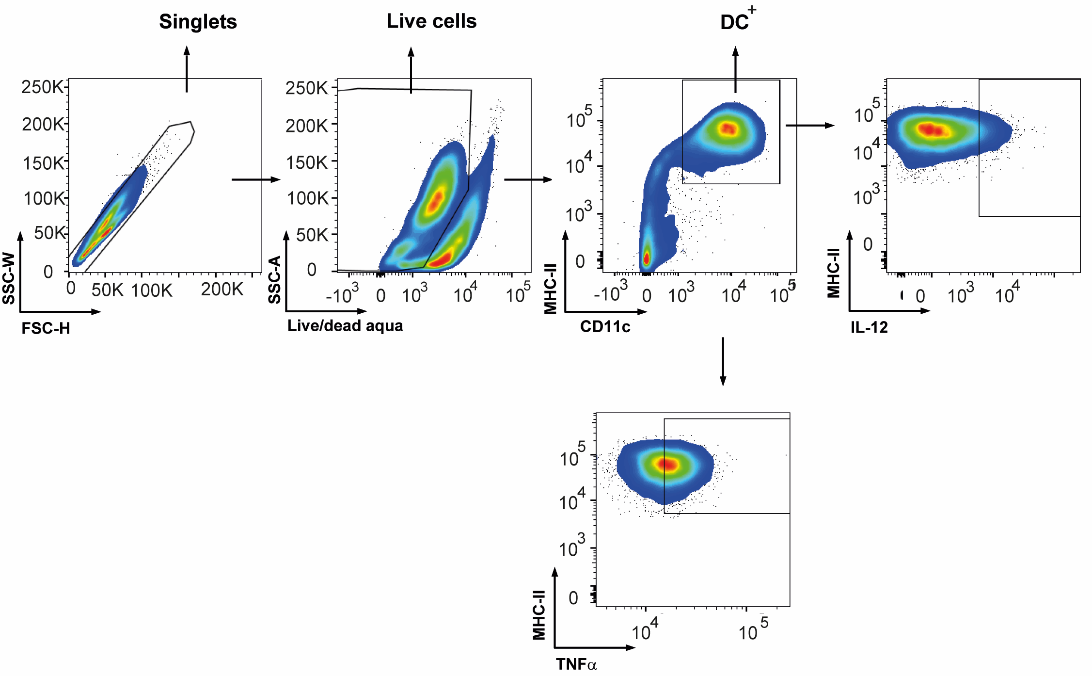
**

**Fig S12.** **Analysis of intracellular cytokines in DC**

Flow cytometry gating strategies to measure intracellular cytokines in DC


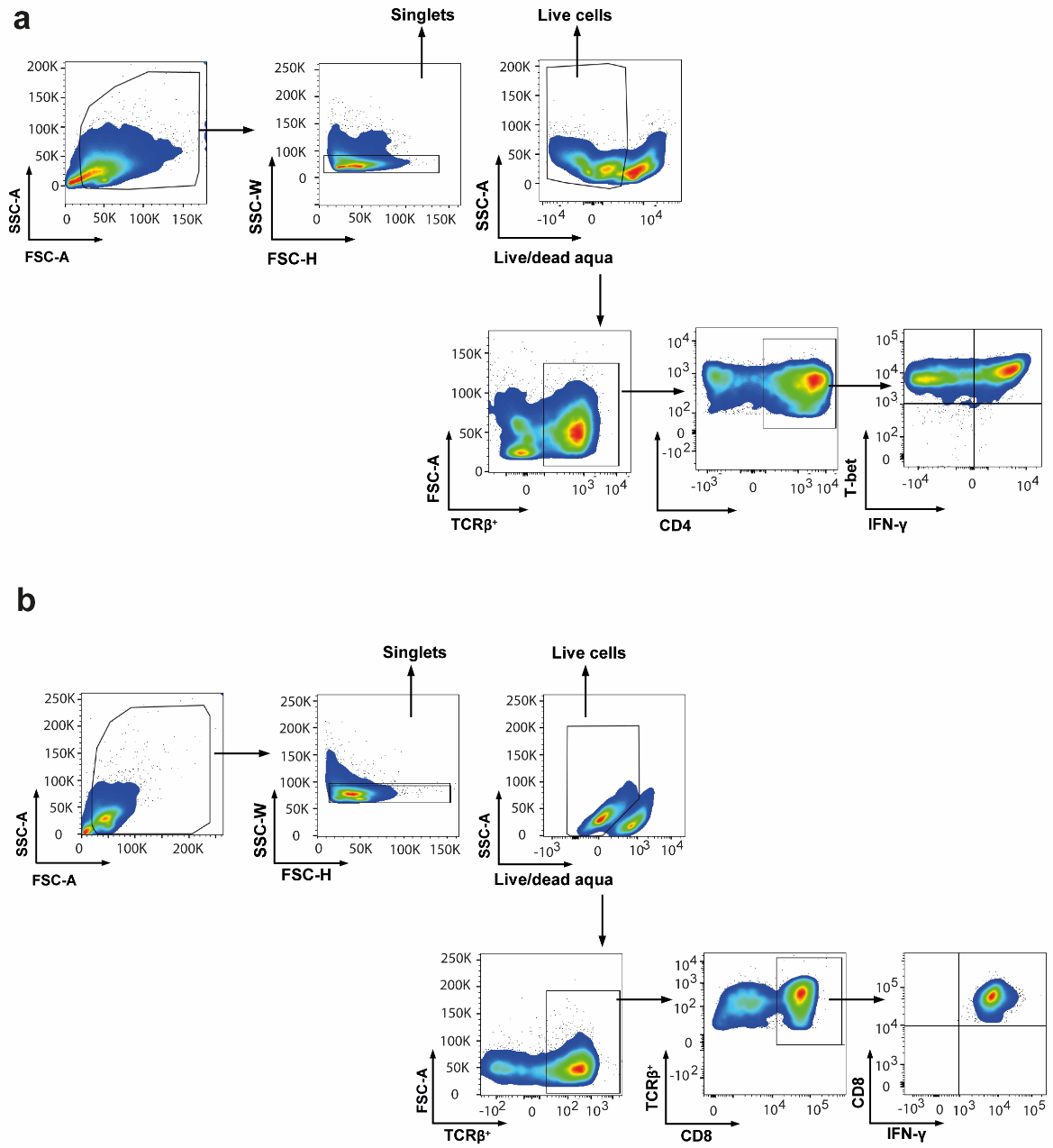


**Fig S13.** **Analysis of CD4 and CD8 T cells activated by DC**

Flow cytometry gating strategies to measure activated CD4^+^ and CD8^+^ T cells by DDA-sEV incubated with sEV
